# Adaptive Whole-Brain Dynamics Predictive Method: Relevancy to Mental Disorders

**DOI:** 10.34133/research.0648

**Published:** 2025-04-05

**Authors:** Qian-Yun Zhang, Chun-Wang Su, Qiang Luo, Celso Grebogi, Zi-Gang Huang, Junjie Jiang

**Affiliations:** ^1^ Key Laboratory of Biomedical Information Engineering of Ministry of Education, School of Life Science and Technology, Institute of Health and Rehabilitation Science, Xi’an Jiaotong University, Xi’an, China.; ^2^ Research Center for Brain-inspired Intelligence, School of Life Science and Technology, Xi’an Jiaotong University, Xi’an, Shaanxi 710049, China.; ^3^ National Clinical Research Center for Aging and Medicine at Huashan Hospital, Fudan University, Shanghai 200433, China.; ^4^ Institutes of Brain Science and Human Phenome Institute, Fudan University, Shanghai 200032, China.; ^5^ School of Psychology and Cognitive Science, East China Normal University, Shanghai 200241, China.; ^6^ Institute for Complex Systems and Mathematical Biology, University of Aberdeen, Aberdeen AB24 3UE, UK.; ^7^ School of Automation and Information Engineering, Xi’an University of Technology, Xi’an, Shaanxi 710048, China.

## Abstract

The Hopf whole-brain model, based on structural connectivity, overcomes limitations of traditional structural or functional connectivity-focused methods by incorporating heterogeneity parameters, quantifying dynamic brain characteristics in healthy and diseased states. Traditional parameter fitting techniques lack precision, restricting broader use. To address this, we validated parameter fitting methods using simulated networks and synthetic models, introducing improvements such as individual-specific initialization and optimized gradient descent, which reduced individual data loss. We also developed an approximate loss function and gradient adjustment mechanism, enhancing parameter fitting accuracy and stability. Applying this refined method to datasets for major depressive disorder (MDD) and autism spectrum disorder (ASD), we identified differences in brain regions between patients and healthy controls, explaining related anomalies. This rigorous validation is crucial for clinical application, paving the way for precise neuropathological identification and novel treatments in neuropsychiatric research, demonstrating substantial potential in clinical neurology.

## Introduction

Neurological and psychiatric disorders have a high prevalence worldwide, including depression, anxiety, schizophrenia, Alzheimer’s disease, and others. These conditions affect hundreds of millions of people and impose a significant social and economic burden. The diagnosis of many of these disorders still relies on subjective clinical assessments and lacks objective biomarkers. Therefore, finding more effective prevention and treatment methods through research is crucial to addressing future health challenges. Currently, research on these disorders mainly focuses on changes in structural or functional network connectivity, revealing variations in the strength and patterns of connections between different brain regions. However, these studies have not sufficiently measured changes in the properties of specific brain regions, limiting a comprehensive understanding of disease mechanisms. In this regard, whole-brain computational models based on structural connectivity (SC) from diffusion MRI as a structural scaffold to simulate mesoscopic neural interactions and functional dynamics can quantify the dynamic characteristics of each brain region in both healthy and diseased states in a highly interpretable manner [1–4]. Subsequently, functional global dynamics emerge from the interactions of local node dynamics. Thus, integrating patient-specific neuroimaging data [such as diffusion tensor imaging (DTI), functional magnetic resonance imaging (fMRI), magnetoencephalography (MEG), electroencephalography (EEG)] with the model, and adjusting parameters at the node, edge, or global level to modulate its biological or phenomenological characteristics, can effectively promote precision therapy [5–9]. The aim of whole-brain modeling is to balance the complexity of the model and its physiological interpretability while describing the brain’s most crucial functional features [10]. Among them, whole-brain computational models originating from statistical physics have been particularly successful, such as global spiking attractor network [11], circuit models [12,13], mean-field models [14,15], dynamic mean-field models [16], linear stochastic model [17], spatial autoregressive model [18], neural mass model [19], Kuramoto model, algebraic models [20], and Hopf model [21–23].

Building on this concept, the normal form of supercritical Hopf bifurcation (Landau–Stuart oscillator) was used to simulate the blood oxygen level-dependent (BOLD) activity for each brain area, based on SC, revealing the impact of anatomical connections on functional dynamics. Currently, many studies are based on a homogeneous approach to brain regions (setting bifurcation parameters at the same value). This is because using a heterogeneous approach results in an excessive number of parameters, making accurate fitting difficult, thus necessitating simplification. Escrichs et al. [24] demonstrated that adjusting the global coupling constant *G* in the model simulates various brain states, including progressively deeper sleep stages, propofol-induced sedation, anesthesia, disorders of consciousness, unresponsive wakefulness syndrome, or minimally conscious state. Perturbation analysis of the model reveals stability differences between states. Incorporating external perturbations as periodic forcing terms helps distinguish brain states based on stability and reversibility [16,25–28].

However, the homogeneous approach fails to adequately reflect the actual heterogeneity of brain regions, which is crucial for studying brain function and structure. Different brain regions have unique structures, functions, and connectivity patterns, which are essential for understanding the overall functionality of the brain. To address this, the heterogeneity parameters $a_{i}$ in the model can quantify the dynamic characteristics of each brain region in both healthy and diseased states in a clear and rational manner. By applying this model to the fit BOLD signals, researchers can establish a mapping relationship between signal characteristics and model parameters, gaining a deeper understanding of the coupling mechanisms and internal dynamic changes among different brain regions. This approach provides novel insights into the understanding of brain functionality and the pathophysiological mechanisms of neurological disorders [21,29,30]. By analyzing changes in bifurcation parameters $a_{i}$ and global coupling constant *G* under different brain states, researchers delve into the mechanisms behind various pathological features and brain state transitions. For instance, compared to wakefulness, *G* significantly decreases in states of diminished consciousness [27,31]. In a normal wakeful state, $a_{i}$ leans more toward negative values. The Hopf whole-brain model also revealed significant functional benefits when combining long-range exceptions with turbulence in information processing [32,33]. Analyzing changes in $a_{i}$ helps identify significant differences in the dynamic characteristics of brain regions across states. For instance, Parkinson’s patients undergoing deep brain stimulation (DBS) exhibit changes in the thalamus and globus pallidus. Regions like the olfactory cortex, amygdala, and hippocampus show enhanced oscillatory behaviors in the DBS off state, weakening in the on state toward an $a_{i}$ value near zero [34]. Moreover, the model plays a crucial role in bridging structural and functional brain connectivity. Tian et al. [35] showed that functional connectivity simulated from structural connections using the MBMv4 atlas aligns closely with empirical data, validating the atlas’s effectiveness.

These studies using the Hopf whole-brain model offer valuable insights into neuropathology, neurorehabilitation, and brain structure–function mapping. However, they emphasize the importance of precise and effective parameter fitting. Before comparing different brain states or pathologies, it is crucial to validate the model’s fitting capabilities and parameter accuracy. Accurate parameters ensure reliable model outputs and confirm the model’s rationality. Only by ensuring precise parameter fitting can we achieve credible, meaningful results in analyzing brain function and its anomalies.

Therefore, this paper conducted extensive simulation validation by generating data through simulated networks and synthetic models. We have innovatively developed a general adaptive whole-brain dynamics predictive method, which also addresses the limitations of traditional methods. This method enables individualized fitting of each subject, significantly improving the model’s performance, applicability, and accuracy. Additionally, we applied this method in practical scenarios, conducting in-depth exploration and analysis. Specifically:

1. To more accurately capture and preserve individual differences in the data, we introduced innovative strategies including personalized initial values, learning rates, and characteristics.
2. We constructed an approximate loss function based on the characteristics of the actual loss function from the synthetic model, effectively evaluating the fitting performance, and identifying the optimal parameter combination.
3. We implemented a gradient adjustment mechanism to improve the fit to individual data features, substantially enhancing the model’s data reconstruction abilities and effectively overcoming the limitations of traditional fitting methods.
4. Through rigorous simulation validation and empirical data analysis, we established that this method significantly enhances the model’s fitting performance and robustness, demonstrating extensive applicability.
5. Utilizing this model and method, we analyzed real datasets of major depressive disorder (MDD) and autism spectrum disorder (ASD), achieving individualized parameter evaluation. This provides quantitative descriptions of dynamic features in different brain regions and reveals significant differences and mechanisms of change between healthy and diseased states.

Our work not only underscores the importance of precise parameter fitting for the model but also offers new methodologies in investigating the neuropathological processes of neuropsychiatric diseases, broadening clinical application prospects.

## Results

**Fig. 1.**
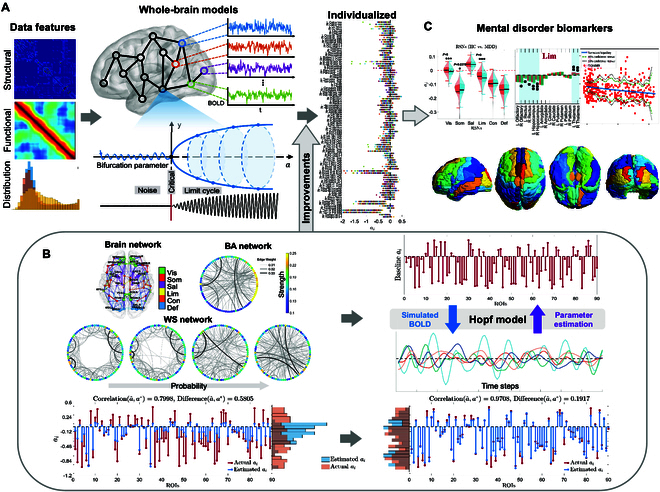
Comprehensive structure of the synthetic model-inspired adaptive whole-brain dynamic prediction method and its application in mental disorders. (A) Model fitting process, where features are extracted from the data to establish a whole-brain network model with nodes that are coupled but have heterogeneous dynamic parameters. Individualized model construction and data reconstruction are achieved through personalized parameter fitting. (B) Significant improvement in fitting performance achieved through the refinement and validation of multiple simulated networks, which include real brain network structures, BA networks, and WS networks with different rewiring probabilities, along with thousands of sets of baseline parameters. Once validated, the process is applied to real data (C). Parameters are fitted for different subjects, and statistical analyses compare parameter differences among groups, expanding the model’s application such as finding the potential biomarkers for mental disorders.

In our work, we corroborated the accuracy of BOLD signal characteristics and parameter fitting, investigating the limitations of fitting performance. Building upon these findings, through extensive experimental validation and simulations, we identified the most suitable initialization method and gradient descent steps based on individual characteristics. Then, we explored the intricate relationship between BOLD signal features and model parameters. This led us to introduce a gradient adjustment mechanism, which significantly reduced information loss and improved the fitting accuracy of individual characteristics. Additionally, we found methods to quantify the correlation and differences in parameter fitting, prompting us to propose an approximate loss function that ensured the accuracy of parameter fitting and the robustness of the model. We also observed that the stability of the fitting system varied across different frequency bands. Our work not only illustrates the effectiveness of our modeling approach but also enhances the understanding of dynamic changes within brain regions under various conditions. Using this modeling approach, we successfully constructed models for 2 large datasets to thoroughly analyze their complex dynamic structures and characteristics: patients with MDD and healthy controls (HCs), as well as patients with ASD and HC. Applying adaptive parameter fitting, we individualized parameter fitting for each subject and compared brain region differences based on medication status, disease condition, gender, and age. We interpreted abnormal brain regions in individuals with mental disorders based on previous research. The analysis process is shown in Fig. 1.

### Limitation identification driven by synthetic models

To validate the effectiveness of the parameter fitting method, we utilize generative models to produce data. First, a set of random parameter vectors $a^{*}={\left\{a_{i}^{*}\right\}}_{i=1}^{N}$ [where i=1,2,…,N, and N represents the total number of regions of interest (ROIs)] is constructed and input into the Landau–Stuart equation of the Hopf whole-brain model to generate synthetic data. The input parameters $a^{*}$, serving as known “ground truth”, are compared with the parameter fitting from the synthetic data to evaluate the accuracy and performance of the parameter fitting method. Through extensive experimental validation and simulation across multiple sets of $a^{*}$, the limitations of the traditional method were identified, and targeted improvements were made.

Building on the simulation framework, comprehensive tests were performed on synthetic model data, covering various network structures and ranges of bifurcation parameters. In the previous approach, all bifurcation parameters were initially homogenized around zero, and the global coupling parameter *G* was optimized based on the fit of functional connectivity dynamics (FCD). Once *G* was determined, regional parameters *p* were used to fine-tune the heterogeneous bifurcation parameters across the brain, yielding a set of heterogeneous parameters $\hat{a}={\left\{{\hat{a}}_{i}\right\}}_{i=1}^{N}$ (N being the number of nodes) along with *G*. Through a series of fitting experiments across various value ranges and network structures, we found that this method reliably determined the sign (positive or negative) of the fitted parameters ${\hat{a}}_{i}$ for each node, ensuring alignment with the corresponding node’s dynamic behavior.

However, the method exhibited significant limitations when applied to quantitative fitting, revealing several challenges in practical applications: Specifically, for $a^{*}$ values around *0*, excessive iterations needed to meet the convergence criterion for *p* resulted in ${\hat{a}}_{i}$ values that significantly exceeded the true values. In contrast, for $a^{*}$ values over a wider range, the method tended to under-fit, as insufficient iterations caused the algorithm to prematurely exit the convergence process, leading to ${\hat{a}}_{i}$ values that were far from the true values. Furthermore, regardless of the parameter range, the method consistently introduced a negative bias in ${\hat{a}}_{i}$. Through extensive testing and analysis, this bias was traced back to errors in the gradient difference calculation during gradient descent. In summary, while the traditional fitting method may perform adequately in certain scenarios, its effectiveness across varying parameter ranges and real fMRI data still leaves considerable room for improvement.

Additionally, previous research methodologies averaged fitting metrics across groups, yielding a single parameter set per group for intergroup comparisons. While this approach captures general trends, it often masks significant individual variability. The key limitation is its inability to thoroughly examine each subject’s unique responses and characteristics, potentially overlooking important individual-level patterns. This averaging process can obscure genuine differences and introduce bias into statistical analyses, leading to conclusions that fail to accurately reflect the cohort. Furthermore, since the goodness of fit for signal characteristics was not used as a constraint, the optimal parameter pairs failed to accurately capture the BOLD signal characteristics. This emphasis on parameter optimization over data accuracy can lead to misleading interpretations, highlighting the need for immediate improvements.

### Method improvements driven by synthetic models

**Fig. 2.**
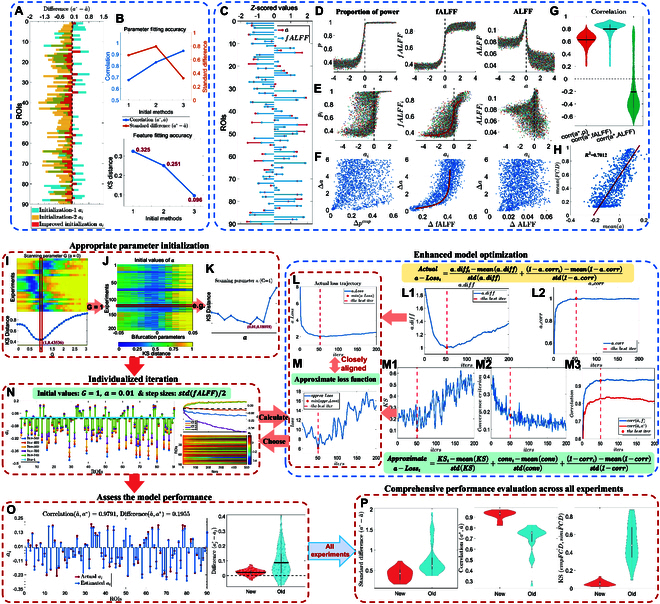
(A) Differences between fitted ${\hat{a}}_{i}$ and actual $a_{i}^{*}$ using 3 initialization methods. (B) Comparison of parameter fitting performance. (C) Relationship between *Z*-score normalized fALFF and a. (D) Simulations without coupling. (E) Simulations with coupling: relationship between a, *p*, fALFF, and ALFF. (F) Relationship between Δa and Δp, ΔfALFF, and ΔALFF, where $\Delta \left(\cdot \right)=\text{max}\left(\cdot \right)-\text{min}\left(\cdot \right)$. (G) Overall correlation comparison [corr(a, fALFF) is highest]. (H) Positive correlation between FCD mean values and *a*. (I to P) Steps of the synthetic model-inspired adaptive parameter fitting method: (I) Optimal *G* values (setting a = 0), with average *G* = 1 as the coupling strength. (J) Optimal homogeneous $\hat{a}$ values (*G* = 1). (K to O) Sample updated via individualized gradient descent. (L) Actual loss function during iterations (minimum at the 53rd iteration), including (L1) difference and (L2) similarity parts. (M) Approximate loss function (minimum at the 52nd iteration), with KS distance (M1), gradient convergence (M2), and similarity (M3). (N) Node variation during iterations, with details of the entire process and individual node updates. (O) Fitting performance of the example. (P) Comparison of all experiments: differences, correlations, and KS distance.

We proposed an adaptive parameter fitting method based on a simulation validation framework using synthetic models, aiming to overcome the limitations of traditional approaches in the Hopf whole-brain model. The overall workflow of the model is illustrated in Fig. 2. After determining the optimal initial values for *G* and the homogeneous $\hat{a}$ through parameter scanning, we proceed to iterate for the heterogeneous parameters ${\left\{{\hat{a}}_{i}\right\}}_{i=1}^{N}$ (“Initialization” section). To prevent information loss from group averaging, we fitted individual parameters based on each subject’s specific features. To further improve parameter fitting accuracy, we employed an evaluation criterion based on fractional amplitude of low-frequency fluctuation (fALFF) convergence to optimize the bifurcation parameters during gradient descent. This criterion ensures scientific rigor and reproducibility (“The characteristic of guiding parameter fitting” section). During the gradient descent process, an adaptive step-size strategy was employed to achieve individualized adjustments. The learning rate was dynamically adjusted based on each subject’s unique data characteristics, specifically the standard deviation of the fALFF indicator. This approach not only improved the efficiency of gradient descent but also significantly enhanced the ability to adapt parameter fitting to individual variations. Additionally, the significant positive correlation between the mean FCD value and mean $\left\{a_{i}\right\}$ provided theoretical support for aligning individualized FCD features through gradient adjustments during the gradient descent process (“Adaptive individualized fitting” section). Finally, based on the simulation results, we proposed an approximate loss function that combines parameter fitting loss and data feature fitting loss. This function approximates the actual loss function, providing a robust evaluation framework that ensures accurate parameter and data fitting (“Loss functions” section). This evaluation system demonstrates exceptional fitting capability and broad generalizability across various contexts.

#### Initialization

Our primary goal is to describe the global spatiotemporal dynamics of each individual in different states, with these dynamic features being independent of changes in local node dynamics. To this end, we investigated network dynamics in a homogeneous scenario, where the bifurcation parameters of all nodes are set to $\hat{a}=0$. This choice was inspired by previous research, which demonstrated that data alignment is most effective at the critical edge of the Hopf bifurcation [22]. In this setup, the dynamic behavior of the network is primarily driven by a single free parameter, the global coupling strength *G*. To fit this parameter, we scanned *G* within the range of 0 to 2 with a step size of 0.1, and computed the Kolmogorov–Smirnov (KS) distance between the empirical FCD and the model-simulated FCD for each parameter value (quantifying the maximum deviation between the cumulative distribution functions of the 2 samples). We then plotted the U-shaped curve of KS values as a function of *G*, where the minimum point of the curve corresponds to the optimal *G* value. Finally, the average of the optimal *G* values across all datasets was calculated to obtain the global coupling strength for the population.

For the individualized initial values of the parameter a, we first tested several initialization strategies and compared their performance, as shown in Fig. 2A and B. After determining the optimal *G* value when $\hat{a}=0$, we employed 3 different initialization methods: Initialization-1 (using scaled fALFF values), Initialization-2 (using a constant value of $\hat{a}=0$), and an improved method that scanned parameters in the range of $\left[-0.05,0.05\right]$ with a step size of 0.01 to determine the optimal homogeneous $\hat{a}$ value. This value was determined by minimizing the KS distance of the fitted FCD features and was used as the initial value for iterating heterogeneous ${\hat{a}}_{i}$ values. Based on the comparative analysis, we selected the initialization method that significantly improved the fitting performance, as shown in Fig. 2I to K. Building upon the individualized initial values, we further extended the model to a heterogeneous one, introducing different bifurcation parameters ${\hat{a}}_{i}$ for each ROI, enabling the assessment of local dynamic heterogeneity within the network.

#### The characteristic of guiding parameter fitting

By introducing the bifurcation parameter $a_{i}$ for different ROIs, we extended the model to a heterogeneous form, enabling a more accurate assessment of local dynamics within the network. $a_{i}$ directly influences the stability of the system. When $a_{i}$ is negative, the oscillator oscillates around a stable fixed point; when $a_{i}$ becomes positive, the fixed point becomes unstable, leading to an increase in the amplitude of periodic oscillations. The oscillation amplitude is proportional to $a_{i}$, emphasizing the direct relationship between the bifurcation parameter and the oscillation amplitude. In previous fitting strategies, the optimization of the heterogeneous bifurcation parameter relied on the low-frequency power ratio $\left(p={\left\{p_{i}\right\}}_{i=1}^{N}\right)$ of each node’s BOLD signal. Specifically, through gradient descent, the parameter $\hat{a}$ is iteratively updated based on the difference between simulated and empirical p, until the difference converges to a predetermined threshold.

In the simulations, we performed 2 sets of 5,000 simulations, analyzing the relationships between 3 indicators—low-frequency power ratio $\left(p={\left\{p_{i}\right\}}_{i=1}^{N}\right)$, fALFF $\left(f={\left\{f_{i}\right\}}_{i=1}^{N}\right)$, and ALFF—and the bifurcation parameter $\left(a={\left\{a_{i}\right\}}_{i=1}^{N}\right)$ after the signal was *Z*-score normalized $\left(\frac{x-\text{mean}\left(x\right)}{\text{std}\left(x\right)}\right)$. In both sets, the constants $\omega ={\left\{\omega_{i}\right\}}_{i=1}^{N}$ and noise intensity were held fixed based on existing literature, with a being set as a variable, represented by a 90-dimensional vector. Each element $a_{i}$ was randomly chosen from a uniform distribution $U\{x_{1},x_{2}\}$, where $x_{1}$ is randomly selected from the range $\left[-4,\;0\right)$ and $x_{2}$ is randomly selected from the range $\left(0,\;4\right]$, and applied to the Hopf whole-brain model. The first set of simulations did not include coupling (Fig. 2D), while the second set maintained a fixed global coupling strength *G* = 1.5 and used actual brain network connections. There is a stronger positive correlation between the parameter a and fALFF values, in terms of node values, means, amplitudes, and the correlation, as shown in Fig. 2E to G, further validating fALFF as an effective metric for fitting $\hat{a}$ in gradient descent.

Through experiments with synthetic data, we further confirmed the effectiveness of the fALFF feature in guiding the fitting of parameter $\left\{{\hat{a}}_{i}\right\}$. In this process, a key normalization step was introduced, where mean scaling was applied to reduce the scale differences between features $\left(\frac{x}{\text{mean}\left(x\right)}\right)$. This method ensures uniform gradient updates across all dimensions during the feature update process, preventing features with larger scales from dominating. The simulation results showed that mean scaling normalization outperforms both *Z*-score normalization and min-max normalization $\left(\frac{x-\text{min}\left(x\right)}{\text{max}\left(x\right)-\text{min}\left(x\right)}\right)$, which preserves the original distribution of fALFF data by dividing by the mean, emphasizing the relative activity levels between different brain regions within an individual. In contrast, parameter fitting focuses on exploring the differences in activity across regions, which are critical for identifying individual-level variations. The *Z*-score and min-max normalization methods tend to scale the data to a unified range, making them more sensitive to outliers. This sensitivity can result in the overemphasis of errors in these regions during the gradient descent process, negatively impacting the model’s accuracy and efficiency. Moreover, the presence of outliers may cause the fALFF values of other ROIs to become overly concentrated after normalization, leading to bias in error calculation and gradient updates, ultimately affecting the overall performance of the model.

#### Adaptive individualized fitting

To preserve individual differences and avoid the loss of critical information due to averaging, we fitted a unique set of parameters for each subject. This personalized parameterization approach provides more precise and detailed data, allowing us to thoroughly analyze the unique responses and characteristics of each subject. This not only enhances the accuracy of the model fitting but also captures subtle variations in the aggregated data that may otherwise be overlooked, offering richer data support for subsequent research and applications.

First, we extracted all data features (such as FCD and fALFF) from each subject and used the FCD feature to fit the initial parameter $\hat{a}$ for each subject. During the subsequent gradient descent process, when updating the heterogeneous parameters $\left\{{\hat{a}}_{i}\right\}$, we applied half of the standard deviation of fALFF as an individualized learning rate, with fALFF serving as the gradient metric and the difference between empirical and simulated fALFF as the initial gradient. Through simulations on the synthetic model, we observed a significant positive correlation between the mean of parameters $\left\{a_{i}\right\}$ and the average FCD value of its generated data (Fig. 2H). Therefore, to optimize the fitting of FCD and better reflect individual differences, we introduced a gradient correction mechanism. Specifically, if the mean simulated FCD during the *t*th iteration was lower than the mean empirical FCD, we applied a positive adjustment to all elements of the initial gradient; conversely, if the mean simulated FCD was higher than the mean empirical FCD, a negative adjustment was applied to all gradient elements:

$$\Delta a_{t}=\;\eta \times (\left(f^{\text{emp}}-f_{t}^{\text{sim}}\right)+\left(\text{mean}\left({\text{FCD}}^{\text{emp}}\right)-\text{mean}\left({\text{FCD}}_{t}^{\text{sim}}\right)\right))$$ (1)

This bias correction method, based on the quality of FCD fitting, significantly improved the model’s fitting accuracy for the data characteristics. Using this approach, parameter optimization reduced the KS distance for FCD fitting to approximately 0.1, demonstrating that this method not only maintains the accuracy of parameter fitting but also significantly improves the fitting quality.

#### Loss functions

During the simulation process, while keeping other parameters constant, we observed that as the bifurcation parameters ${\left\{a_{i}\right\}}_{i=1}^{N}$ increased or decreased, the corresponding indicators ${\left\{f_{i}\right\}}_{i=1}^{N}$ exhibited a trend of converging toward a fixed value, as shown in Fig. 2E, with the convergence following a sigmoid function form. Traditional parameter fitting methods perform gradient descent iterations until the maximum difference between the empirical and simulated indicators falls below a set threshold r, i.e., when $\eta =\text{max}\left(\left|f^{\text{emp}}-f_{t}^{\text{sim}}\right|\right)<r$, the iteration stops, and the corresponding parameter ${\hat{a}}_{t}$ is considered optimal. However, due to the sigmoid-like relationship between parameters and indicators, the gradient error rapidly decreases during the iterations but enters a fluctuating convergence state, with an unstable convergence value (as shown in Fig. 2M2). As a result, selecting an appropriate gradient descent threshold r is challenging, and the effectiveness of parameter fitting is highly sensitive to this choice. If r is set too large, it may lead to insufficient parameter iteration, deviating from the target value. On the other hand, if r is set too small, the iteration may take too long or fail to meet the convergence criterion. Additionally, we observed that during the stage when the error fluctuates near the convergence value, gradient updates are concentrated on only a few specific nodes, and no matter how the bifurcation parameters are updated, the error at these nodes remains almost unchanged. Through extensive experiments, we realized that solely relying on error convergence to determine iteration termination is insufficient. The true challenge lies in selecting an appropriate convergence threshold, as this choice significantly impacts the model’s fitting quality.

#### Actual loss function

To more accurately determine whether the iterations of gradient descent have reached the target value, we introduced a loss function as the evaluation criterion. In our experiments, the value of $\left\{a_{i}\right\}$ for the generated data is known (representing the true bifurcation parameter value). In this context, the actual loss function can be used to assess the goodness of fit of our fitting method, as well as to validate the performance of the simulated parameters in terms of both quantitative (difference) and qualitative (correlation) aspects. Therefore, the actual loss function consists of 2 parts: the numerical difference in fitting ($a\_{\text{diff}}_{t}=\text{mean}\left(\left|a^{*}-{\hat{a}}_{t}\right|\right)$, Fig. 2L1) and the fitting similarity ($a\_{\text{corr}}_{t}=1-\text{corr}\left(a^{*},\;{\hat{a}}_{t}\right)$, Fig. 2L2), where $a^{*}={\left\{a_{i}^{*}\right\}}_{i=1}^{N}$ represents the actual value of bifurcation parameters; ${\hat{a}}_{t}$ is the fitted parameter value in iteration t (t=1,…,I). To ensure that both components contribute equally to the overall loss, thereby providing a more comprehensive and accurate assessment of the model’s performance, we converted each component’s loss value into a proportion of deviation relative to its best performance during the iteration process. The calculation formulas for these 2 components are defined as follows (Fig. 2L):

$$\text{Actual}\;a-{\text{Loss}}_{t}=\frac{a\_{\text{diff}}_{t}-\text{mean}(a\_\text{diff})}{\text{std}(a\_\text{diff})}+\frac{a\_{\text{corr}}_{t}-\text{mean}(a\_\text{corr})}{\text{std}(a\_\text{corr})}$$ (2)

#### Approximate loss function

To effectively fit parameters $\left\{a_{i}^{*}\right\}$ in actual data settings, we confronted a challenge: The actual values of these parameters were unknown, making it impossible to directly use them as a benchmark for evaluating the fitting performance. Hence, we have devised an approximate a-Loss function.

For quantifying the fitting performance, this approximate loss function was divided into 2 parts. The first part was based on the fitness quality of the FCD. We utilized the simulated parameter values ${\hat{a}}_{t}$ from each iteration to compute the simulated FCD and assessed its divergence from the actual FCD using the KS distance, thereby evaluating the current parameters’ fitting adequacy to the data features: ${\mathrm{KS}}_{t}$, as shown in Fig. 2M1). The second part pertained to the convergence of the fALFF indicator as observed through gradient descent. Specifically, we inserted the simulated parameter values obtained in each iteration into the dynamical equation, calculating the corresponding simulated fALFF values ($f_{t}^{\text{sim}}$). We then computed their differences with the actual fALFF values ($f^{\text{emp}}$) at each ROI, and the maximum absolute value of these differences serves as a quantitative measure of parameter convergence (see Fig. 2M2): ${\text{conv}}_{t}=\text{max}\left(\left|f^{\text{emp}}-f_{t}^{\text{sim}}\right|\right)$. Regarding the qualitative fitting performance, our simulations revealed a strong correlation between a and f. Excessive gradient descent may lead to parameter values for nodes in the asymptotic regions exceeding their target values, significantly reducing the correlation between the parameter vector and the fALFF features. Further validation showed that the correlation between the simulated parameter $\hat{a}$ and the actual parameter $a^{*}$ $\left(\text{corr}\left({\hat{a}}_{t},\;a^{*}\right)\right)$ was consistent with the correlation between $\hat{a}$ and $f^{\text{emp}}$ throughout the gradient descent iterations (see Fig. 2M3). This implied that, in the absence of known actual parameter values, we can use the correlation between the simulated parameters and the empirical features as part of the loss function to assess the qualitative fitting performance: ${\text{corr}}_{t}=\text{corr}\left({\hat{a}}_{t},\;f^{\text{emp}}\right)$. After *Z*-score standardization, the approximate loss function is represented as:

$$\text{Approximate}\;a-{\text{Loss}}_{t}=\frac{KS_{t}-\text{mean}(KS_{t})}{\text{std}(KS_{t})}+\frac{{\text{conv}}_{t}-\text{mean}({\text{conv}}_{t})}{\text{std}({\text{conv}}_{t})}+\frac{{\text{corr}}_{t}-\text{mean}({\text{corr}}_{t})}{\text{std}({\text{corr}}_{t})}$$ (3)

To validate the efficacy of the approximate loss function, we conducted tests on generated data. The results revealed a high degree of similarity in performance between the actual loss function and the approximate loss function during the gradient descent. By comparing the minimum value points of these 2 loss functions, we were able to consistently identify the iteration that most closely approached the target value. For example, as shown in Fig. 2L and M, the actual loss function points to the 53rd iteration as the optimal fit, while the approximate loss function points to the 52nd iteration. The results are similar, with the parameters ${\hat{a}}_{52}$ being the optimal ones. This indicates that the designed approximate loss function is a reliable and effective tool for evaluating the quality of parameter fitting.

To comprehensively evaluate the effectiveness of this method, we conducted extensive simulation experiments to validate its performance and compared the fitting results before and after the method’s improvement. The results demonstrated significant enhancements in fitting performance, particularly achieving systematic improvements in parameter fitting accuracy, correlation, stability, and data feature fitting goodness, as shown in Fig. 2O and P. Specifically, parameter fitting stability improved by 38.94%, as evidenced by a notable reduction in standardized differences; correlation with the true parameters increased by 22.58%; and the accuracy of data feature fitting improved by 82.51%, which was validated by the reduction in KS distance.

Additionally, we verified the generalizability of this method, demonstrating that it is not only applicable to the current network size but can also be extended to other brain templates of varying scales, such as the power template with 264 nodes (see Fig. S1). However, as the granularity of brain parcellation increases, the complexity of the template and the associated computational cost also grow significantly.

### Fitting performance across different frequency bands

**Fig. 3.**
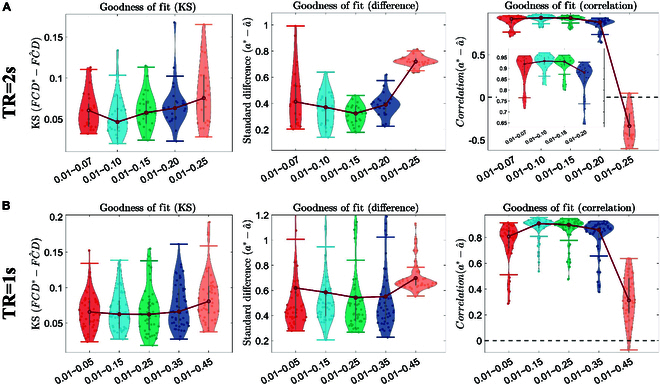
The fitting performance of the adaptive Hopf whole-brain model parameter fitting method across frequency bands is as follows: (A) TR = 2s (Nyquist frequency = 0.25Hz), displaying the fitting performance for fluctuation proportions across different bands relative to the 0.01 to 0.25Hz band. (B) TR = 1s (Nyquist frequency = 0.5Hz), illustrating the fitting outcomes for fluctuation proportions relative to the 0.01 to 0.5Hz band. The left, middle, and right graphs respectively show the fitting goodness (KS value) of the FCD matrix, the fitting differences, and the correlation of parameter $\hat{a}$.

In our numerous experiments, each experiment involved generating random parameter set to investigate the impact of frequency band range selection on fitting performance. The results indicated that as the frequency band range expanded, the fitting initially improved. However, when the range was further increased to cover the full spectrum, the fitting performance began to decline, which was demonstrated in Fig. 3. Optimal fitting performance was observed under conditions close to half of the full frequency range, where the correspondence between the fALFF feature and parameter a was most pronounced. This suggests that fitting within a specific range of frequency bands can yield better results. Conversely, fitting over too broad or too narrow a frequency range may introduce additional interference, thereby affecting the model’s accuracy and effectiveness.

### Application to the REST-meta-MDD data

**Fig. 4.**
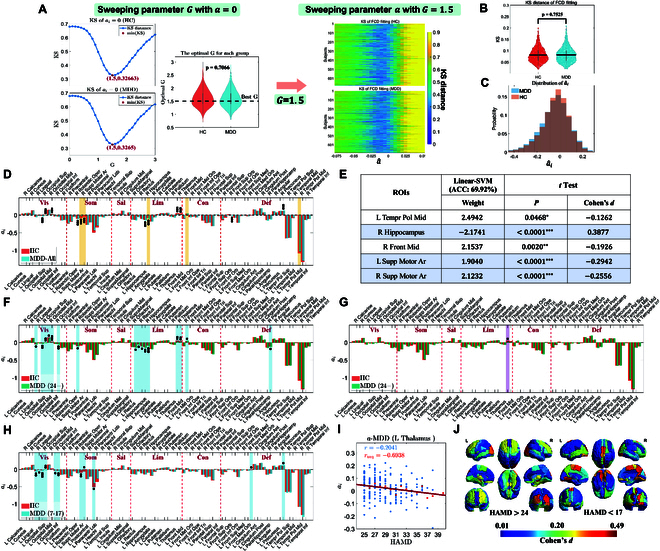
(A) Initial value selection: a is set to 0, followed by parameter scanning to determine the optimal global coupling parameter (G=1.5) and homogeneous $\hat{a}$ for each subject [HC (648) and MDD (666)]. (B) Fitting performance (KS distance) of heterogeneous $\left\{{\hat{a}}_{i}\right\}$ shows no significant difference between groups (*t* test, P=0.7525), ensuring unbiased subsequent analyses. (C) Distribution of heterogeneous $\left\{{\hat{a}}_{i}\right\}$ parameters across groups. (D) Average bifurcation parameters $\left\{{\hat{a}}_{i}\right\}$ for each ROI in the HC and MDD groups. Asterisks indicate significant differences (*t* test: P<0.05, Cohen’s d>0.25), with significance levels denoted as ${}^{*}$(0.01<P<0.05), ${}^{**}$(0.01<P<0.001), and ${}^{***}$(P<0.001). Shaded regions highlight the top 5 ROIs with the highest linear-SVM weights. (E) Classifier weights for key ROIs with *t* test statistics, where shaded areas indicate significant weights and statistical differences. (F to J) Relationships between $\left\{{\hat{a}}_{i}\right\}$ and HAMD scores: (F) *t* test results showing significant differences between severe MDDs and HCs after FDR correction; (G) associations between HAMD scores and $\left\{{\hat{a}}_{i}\right\}$ in severe MDDs (LMM, FDR-corrected); (H) significant *t* test differences between mild MDDs and HCs after FDR correction; (I) variation of $\left\{{\hat{a}}_{i}\right\}$ in the left thalamus with HAMD scores (entire dataset: r=−0.2041, averaged data: $r_{\mathrm{avg}}=-0.6938$); (J) visually compares Cohen’s *d* effect sizes for differences between MDD and HC groups.

Based on the adaptive Hopf whole-brain model parameter fitting method described above, we initially set the homogeneous parameter to a=0 and performed parameter scanning to determine the optimal global coupling parameter, G=1.5, for each participant. With G=1.5 fixed, the initial $\hat{a}$ was scanned uniformly from −0.075 to 0.05 with a step size of 0.005. The KS values were calculated, and the $\hat{a}$ corresponding to the minimum KS value was selected as the optimal parameter. Using these initial values, we applied the adaptive method to obtain heterogeneous bifurcation parameters $\left\{{\hat{a}}_{i}\right\}$ for 666 MDD patients and 648 HC subjects [36,37]. Combined with the parameter G, these bifurcation parameters, constrained by the approximate loss function, approximated the target values, effectively fitting BOLD data features and revealing dynamic characteristics of brain regions across different pathological states. For example, in fitting FCD matrices, the average KS distance was $0.0820\;\left(\mathrm{SD}=0.0319\right)$ for the MDD group and $0.0812\;\left(\mathrm{SD}=0.0307\right)$ for the HC group, as shown in Fig. 4B, demonstrating satisfactory fitting performance. To analyze group differences in $\left\{{\hat{a}}_{i}\right\}$, we conducted a *t* test on the KS fitting indices of the HC and MDD groups to ensure that the differences were not due to variations in fitting performance. Results indicated no significant difference in goodness of fit between the 2 groups (P=0.7525, as shown in Fig. 4B).

#### Classifications

To explore the application of $\left\{{\hat{a}}_{i}\right\}$ in MDD classification, we divided MDD patients into subgroups based on individual disease information and conducted 10 classification experiments. In each experiment, $\left\{{\hat{a}}_{i}\right\}$ vectors from each subject were used as input features for both linear and radial basis function (RBF) kernel support vector machines (SVMs). Model hyperparameters were selected according to best practices in the literature [38] and evaluated using GridSearchCV, a hyperparameter estimator from the Scikit-learn package. This approach assessed all possible parameter combinations during cross-validation to identify the optimal set. After determining the best hyperparameters, the SVM model was retrained on the full training dataset, and its performance was evaluated on the test dataset. Key performance metrics, including accuracy, precision, recall, and F1 scores, were computed. Using a 5-fold cross-validation strategy, the mean and standard deviation of these metrics were calculated. The final classification results are summarized in Table 1 and Table S1. Table 1 compares classification accuracy between medicated and non-medicated MDD groups using either functional connectivity (FC) matrix or $\left\{{\hat{a}}_{i}\right\}$ as features within the same classifier. The results indicate that bifurcation parameters generally provide superior classification performance. Table S1 presents additional classification details for various MDD subgroups, with participant numbers detailed in Materials and Methods.

**Table 1.** The classification performance of MDD-HC

Contrast	Model	Precision (%)	Recall (%)	F1 (%)	Accuracy (%)	Accuracy FC (%) [39]
MDD vs. HC	linear-SVM	70.31 (4.60)	64.84 (4.54)	67.44 (4.39)	69.92 (2.92)	60.39
	rbf-SVM	70.98 (2.82)	68.95 (3.47)	69.88 (2.22)	70.27 (2.57)	63.15
MDD nonMed vs. HC	linear-SVM	69.95 (2.49)	79.44 (4.63)	74.30 (2.38)	72.70 (1.39)	60.90
	rbf-SVM	79.93 (4.76)	86.02 (3.74)	82.77 (3.39)	81.89 (4.59)	60.87
MDD Med vs. HC	linear-SVM	76.81 (2.81)	86.33 (3.08)	81.29 (2.90)	80.10 (3.47)	65.15
	rbf-SVM	87.99 (2.76)	91.03 (2.84)	89.46 (2.34)	89.25 (2.58)	65.80
MDD nonMed vs. MDD Med	linear-SVM	60.46 (10.40)	56.52 (6.66)	57.73 (5.14)	59.95 (4.00)	56.57
	rbf-SVM	93.31 (2.02)	90.49 (3.58)	96.54 (0.90)	65.59 (6.66)	60.99

To analyze the classification characteristics, we employed a linear-SVM approach, which not only achieves classification accuracy comparable to other methods but also enables direct extraction of feature importance from its weights. For all participants, linear-SVM was used to rank key features, with the top 5 most important brain regions highlighted in the shaded areas of Fig. 4D and E. In groups with moderate classification performance, we observed a high degree of consistency between feature weights and statistical significance. During the classification of HC and MDD, linear-SVM achieved a classification accuracy of 69.92% (precision = 70.31%, recall = 64.84%, F1 = 67.44%). Further analysis revealed that the $\left\{{\hat{a}}_{i}\right\}$ values of the top 5 ROIs based on feature weights exhibited significant differences between the MDD and HC groups in the *t* test, with results further validated by false discovery rate (FDR) correction (P<0.05). Additionally, by incorporating effect size (Cohen’s *d*, d>0.25) into our criteria, we identified 11 significant ROIs (see Fig. 4D). Among these, 3 of the top 5 ROIs showed strong significance in both statistical measures and effect sizes, including the R Hippocampus (P<0.0001, d=0.3877), L Supp Motor Area (P<0.0001, d=−0.2942), and R Supp Motor Area (P<0.0001, d=−0.2556). These ROIs hold promise as potential biomarkers for distinguishing HC and MDD.

In classification experiments comparing different MDD groups and HC, we found that in groups with high classification performance, the top-ranked ROIs in classifier weights closely aligned with ROIs showing significant differences in *t* tests and large Cohen’s *d* effect sizes. This indicates that these key ROIs demonstrate strong discriminatory power and reliability in distinguishing health and disease states, providing valuable insights for understanding and differentiating these conditions. For instance, in the classification of first episode medicated (FEMed) MDD and HC, the classification accuracy reached 83.81% (precision = 80.16%, recall = 89.92%, F1 = 84.64%), with key differences observed in ROIs such as L Temporal Mid, R Supp Motor Area, R Front Sup Orb, R Temporal Mid, and L Supp Motor Area (see Fig. S2A). Similarly, in the classification of recurrent MDD and HC, accuracy reached 81.75% (precision = 78.77%, recall = 87.36%, F1 = 82.66%), with critical biomarkers identified in R Angular, R Rolandic Oper, R Supp Motor Area, R Front Med Orb, and L Supp Motor Area (see Fig. S2C). These significant differences in specific ROIs across MDD conditions may reflect their distinct pathophysiological characteristics. In the classification and *t* test analysis of 10 different MDD conditions, we observed relatively low classification accuracy for FEMed and first episode drug-naïve (FEDN) MDD groups, at 60.06% and 58.20%, respectively (see Fig. S3A and B). No statistically significant ROIs were detected in these comparisons. However, classification accuracy between first-episode and recurrent MDD was significantly higher, reaching 72.09%. Both the classifier and statistical tests consistently identified significant differences in R Angular, R Rolandic Oper, and L Caudate (see Fig. S3C).

Furthermore, we found that the rbf-SVM classifier generally outperformed linear-SVM in most experiments. For the comparison between HC and all MDD participants, the classification accuracy reached 70.27%. In comparisons between FEMed MDD and HC, FEDN MDD and HC, recurrent MDD and HC, and first-episode and recurrent MDD, the accuracy exceeded 85.00% (specifically 95.88%, 87.47%, 93.31%, and 88.49%, respectively, as shown in Table S1). These results demonstrate that bifurcation parameters, as selected classification features, effectively characterize differences between MDD and HC, and can reliably identify brain regions associated with depression.

#### HAMD score association

We also analyzed the association between bifurcation parameters $\left\{{\hat{a}}_{i}\right\}$ fitted for MDD and HC subjects and the Hamilton depression rating scale (HAMD) scores using a linear mixed-effects model (LMM). The widely accepted HAMD scoring criteria are as follows: a total score of <7 indicates normal; scores between 7 and 17 suggest potential depression; scores between 17 and 24 confirm depression, while scores above 24 indicate severe depression [40]. Within the overall range of depressive scores (HAMD > 7), no significant association was observed between any ROI’s bifurcation parameters and the severity of symptoms. Therefore, based on the established HAMD scoring criteria, we subdivided the 666 MDD participants into 3 scoring groups: 7 to 17 points (114 MDDs), 17 to 24 points (348 MDDs), and >24 points (204 MDDs). Among these groups, a significant negative correlation was found between bifurcation parameters of the Left Thalamus and HAMD scores in the severe depression group (HAMD >24). Importantly, this correlation was independent of other variables and remained significant after correcting for the FDR across 90 ROIs (LMM: P=0.0373, T=−2.8350, r=−0.2041 based on the entire dataset, $r_{\mathrm{avg}}=-0.6938$ based on averaged data for each score point, FDR-corrected). Additionally, these $\left\{{\hat{a}}_{i}\right\}$ values were significantly different from those of the HC group (*t* test: P=0.0141, Cohen’s d=0.2577, FDR-corrected), as shown in Fig. 4F. This finding underscores the critical role of the thalamus in MDD. The detailed trend of ${\hat{a}}_{i}$ values of the left thalamus with changing HAMD scores is illustrated in Fig. 4I.

To further investigate the differences in dynamic brain characteristics between mild and severe MDD patients and HC, we performed a comprehensive ROI comparison using FDR-corrected *t* tests. The results revealed that patients with more severe MDD exhibited greater significant differences in nodes within the limbic network and frontal regions, accompanied by larger effect sizes, particularly in the rectus, hippocampus, and thalamus. Notably, the limbic system plays a crucial role in emotion regulation, memory formation, and stress response. These findings highlight the potential link between severe depression and dysfunction in the limbic system. To provide a more intuitive presentation of the results, Fig. 4F and H highlight brain regions with significant differences between mild and severe MDD groups compared to HC after *t* tests and FDR correction. Additionally, Fig. 4J visually compares the effect sizes of differences among mild and severe MDD groups and HC, effectively illustrating the variations across brain regions and offering a comprehensive perspective on these differences.

### Application to the ABIDE ASD data

**Fig. 5.**
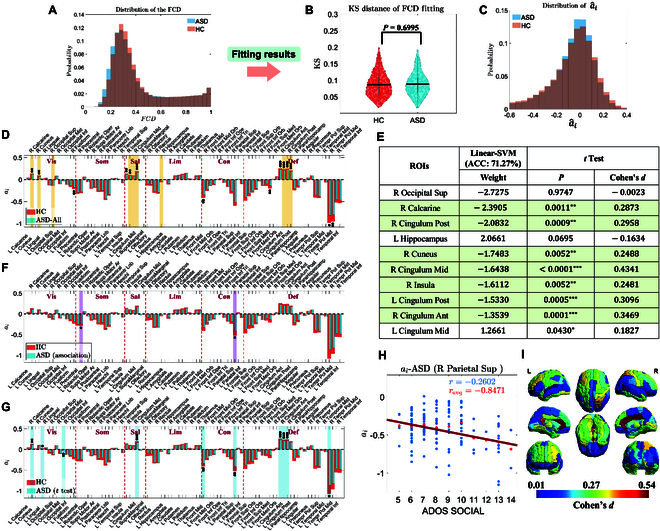
(A) The distribution of FCD matrices from BOLD data reveals minor differences between HC(412) and ASD(332) groups. (B) No significant difference is observed in the final fitting performance (KS distance) of heterogeneous $\left\{{\hat{a}}_{i}\right\}$ between the 2 groups (*t* test, P=0.6995), with all average KS values below 0.1. (C) Distribution of $\left\{{\hat{a}}_{i}\right\}$ across both groups. (D) ROIs marked with asterisks show statistically significant differences and a medium effect size (*t* test: P<0.05, Cohen’s d>0.25). Shaded areas highlight the top 10 ROIs with the highest linear-SVM weights. (E) Weights of key classifier nodes and corresponding *t* test results, with shaded areas denoting ROIs of substantial classifier weights and statistical significance. (F) LMM results show significant associations between ADOS-Social score and $\left\{{\hat{a}}_{i}\right\}$ after FDR correction. (G) *t* Test results reveal significant differences between ASDs and HCs after FDR correction. (H) Variation in $\left\{{\hat{a}}_{i}\right\}$ values in the right SPG as scores change (entire dataset: r=−0.2602; averaged data at each score point: $r_{\mathrm{avg}}=-0.8471$) ($0.01<P<0.05:{}^{*}$, $0.01<P<0.001:{}^{**}$, $P<0.001:{}^{***}$). (I) Effect size (Cohen’s *d*) visually compares differences between ASDs and HCs.

First, we observed subtle differences in the FCD distribution between the HC and ASD groups (as shown in Fig. 5A). Subsequently, we applied the adaptive parameter fitting method, starting with the homogeneous model to preliminarily fit the global coupling parameter *G* and the homogeneous parameter $\hat{a}$. We then successfully fitted the heterogeneous parameter $\left\{{\hat{a}}_{i}\right\}$ for 332 ASD and 412 HC subjects [41,42]. These parameters achieved an optimal fit to the BOLD signal characteristics, effectively capturing the dynamic features of brain regions for each participant. In the ASD group, the average KS distance for the fitted FCD was 0.0880(SD=0.0346), while in the HC group, it was 0.0868(SD=0.0358). No significant difference in fitting accuracy was observed between the 2 groups in the *t* test (P=0.6995, as shown in Fig. 5B). Finally, the distribution of brain region parameters across both groups (Fig. 5C) showed a more pronounced peak in the ASD group, with a probability distribution concentrated closer to a=0. In contrast, the parameter distribution in the HC group exhibited greater dispersion.

#### Classifications

We divided the ASD subjects into different groups based on their non-imaging information (group sizes provided in Table 3) and conducted classification experiments using the same methods and classifiers as those applied to the MDD dataset. The raw bifurcation parameters *a*, without any special processing, were directly used as features after *Z*-score normalization and input into the SVM classifier. Key performance metrics, including accuracy, precision, recall, and F1 score, were used to evaluate classification performance, confirming the reliability of these parameters. Detailed results are presented in Table 2, which compares classification accuracy using FC features in the same classifier, and Table S2. Moreover, this straightforward approach demonstrated certain advantages in classification accuracy over more complex methods, such as graph convolutional neural networks, which often require extensive data adjustments and the integration of non-imaging and structural information, as shown in Table S4.

**Table 2.** The classification performance of ASD-HC

Contrast	Model	Precision (%)	Recall (%)	F1 (%)	Accuracy (%)	Accuracy (%) with FC [43]
ASD vs. HC	linear-SVM	69.03 (6.79)	73.40 (2.74)	71.03 (4.51)	71.27 (3.65)	68.86
	rbf-SVM	73.52 (3.39)	75.35 (6.75)	74.36 (4.77)	75.90 (4.40)	69.43

Following classification, we first analyzed nodal features using a linear-SVM with observable classification weights. We identified the top 10 most significant brain region nodes in the ASD versus HC classification, as indicated by the shaded areas in Fig. 5D and E. Further, we conducted intergroup *t* tests and FDR corrections, followed by calculating Cohen’s d values to quantify the effect size. Nodes with significant differences and at least a medium effect size (P<0.05 and d>0.25) are marked with asterisks in Fig. 5D. Notably, among the 10 ROIs, 7 not only featured in the top 10 classification weights but also exhibited significant statistical and effect size results [e.g., R Calcarine (P=0.0011, d=0.2873), R Posterior Cingulate Gyrus(PCC) (P=0.0009, d=0.2958), R Cuneus (P=0.0052, d=0.2488), R Median Cingulate and Paracingulate Gyri(MCC) (P<0.0001, d=0.4341), R Insula (P=0.0052, d=0.2481), L Posterior Cingulate Gyrus(PCC) (P=0.0005, d=0.3096), and R Anterior Cingulate and Paracingulate Gyri(ACC) (P<0.0001, d=0.3469)], as shown by the overlap of stars and shaded areas in Fig. 5D. These ROIs hold promise as biomarkers for differentiating ASDs from HCs. In the classification tasks of other subgroups, the key feature nodes identified using the same method remained largely the same with minimal variation. This finding suggests that these brain regions, as biomarkers for distinguishing between ASD and HC, exhibit high stability and effectiveness. For detailed information, please refer to Fig. S4.

#### ADOS-Social score association

We continued to use LMMs to investigate the correlation between $\left\{{\hat{a}}_{i}\right\}$ of different brain regions and autism diagnostic observation schedule (ADOS) scores. The ADOS-Social score, a critical component of the ADOS scale, is primarily used to evaluate an individual’s ability in social interactions. By assessing performance in social responsiveness, non-verbal communication, and the establishment and maintenance of social relationships, this score reveals deficiencies in social skills. Our findings demonstrated a significant correlation between ${\hat{a}}_{i}$ of the right superior parietal gyrus (SPG) (R Parietal Sup) and the ADOS-Social score, independent of covariates. Significant differences were also observed between the ASD and HC groups, with an effect size greater than 0.25 (LMM: P=0.0236, T=−3.5521, r=−0.2602 based on the full dataset, $r_{\mathrm{avg}}=-0.8471$ based on the averaged data at each score point, with FDR correction; *t* test: P=0.0054, Cohen’s d=−0.3734, with FDR correction). As shown in Fig. 5F and G, these findings underscore the critical role of this brain region in ASD. Additionally, Fig. 5H illustrates the trend of bifurcation parameter values in the Parietal Sup as the ADOS-Social score changes.

## Discussion

In this work, we utilized the Landau–Stuart equation to simulate whole-brain BOLD dynamics and develop a validation framework based on synthetic data. This led us to devise an adaptive Hopf whole-brain model parameter fitting method. We demonstrated that this adaptive method not only accurately predicts the FCD distribution but also can precisely fit bifurcation parameters ${\left\{{\hat{a}}_{i}\right\}}_{i=1}^{N}$ for any brain network structure and distribution pattern. We then applied this robust method to the BOLD signal datasets of MDD and ASD, fitting ${\left\{{\hat{a}}_{i}\right\}}_{i=1}^{N}$ of each subject. These parameters, used as classification features, demonstrated excellent performance in distinguishing different mental disorders from HCs. We also conducted hypothesis testing, identified potential biomarkers, and performed an in-depth analysis and discussion based on their parameter characteristics.

### Potential biomarker and related previous work for MDDs

By analyzing the bifurcation parameter of each brain region, we found that R Hippocampus, L Supp Motor Ar, and R Supp Motor Ar were statistically significant and also showed prominent effect sizes. Moreover, these areas had considerable weights in the linear-SVM classifier, which could be potential biomarkers to distinguish between HCs and MDDs.

Specifically, we found that in patients with depression, a more negative bifurcation parameter in the Hippocampus indicated that the activity level of this region was lower and less susceptible to change in response to external or internal stimuli. As a pivotal region in the brain responsible for memory, learning, and emotional regulation, the hippocampus’s reduced activation levels could manifest as functional inhibition, which may impair its normal regulation of emotional and cognitive processes, potentially triggering symptoms of depression. We infer that this phenomenon may be due to atrophy or a reduction in volume of the brain area. In fact, numerous MRI and fMRI studies have confirmed that MDD patients exhibit significant reductions in gray matter volume and altered functional brain responses to emotional stimuli in the hippocampus, which are linked to impaired processing of negative emotion and cognitive functions [44–47].

Compared to HCs, we found that the bifurcation parameter of the supplementary motor area (SMA) in MDDs increased from a negative value toward zero (from a relatively stable state to critical point), being more susceptible to environmental or internal changes, and higher activation. Actually, higher activation in the SMA of MDDs suggests that increased neural activity within the SMA is specifically associated with psychomotor retardation in depression [48,49], and this condition can be alleviated via inhibitory repetitive transcranial magnetic stimulation (rTMS) over the SMA [50]. Moreover, through network simulation validation, we found that during this type of transition, the changes in FC between this region and others exhibited uncertainty and could be non-monotonic near the critical point. As we all know, the SMA plays a critical role in controlling and preparing movements, learning and executing motor sequences, emotional processing, and linking internal sensory cues to external rewards for specific actions [51,52]. Therefore, we speculate that this shift may lead to instability and FC dysfunction in the SMA, potentially causing unsteadiness in motor control, emotional expression, and other behavioral outputs. Numerous studies support our hypothesis; for example, disruptions in SC-FC coupling within the SMA have been linked to cognitive deficits in MDDs [53]. In our inference, diminished SC-FC coupling might undermine neural network stability, thereby affecting the cognitive and motor response capabilities of the SMA. Additionally, Zhang et al. [54] discovered that enhanced FC from the occipital cortex (OCC) to the right SMA influences changes in global FCD in MDD. Decreased connectivity in the primary sensory cortices, right SMA and bilateral thalami [55], as well as between the SMA and basal ganglia in MDDs has been reported [56]. Marten et al. [57] linked motor performance deficits in bipolar depression to disrupted connectivity between the posterior cingulate cortex and the SMA. In our understanding, these alterations could correlate with an increased sensitivity to environmental or internal changes, potentially leading to instability in behavioral outputs.

Furthermore, our detailed analysis of bifurcation parameters revealed a significant correlation with the severity of MDD symptoms, emphasizing the crucial role of the Thalamus, which serves as an essential relay station in the brain, responsible for transmitting various sensory information and regulating emotional and higher-order cognitive functions [58,59]. We found that as HAMD scores increased, the bifurcation parameter of the thalamus gradually decreased from positive values toward zero or even negative values, indicating a transition from stable limit cycle oscillation mode to a more unstable critical state or inhibited noisy oscillation mode. This suggests a decrease in the intrinsic activation level or driving force of the thalamus as MDD symptoms worsen. This was also reported by Xue et al. [60] that MDDs had decreased entropy in the bilateral thalami compared with HCs, potentially due to thalamic atrophy. Supporting this, Lu and colleagues [61,62] found significant volume reductions and morphological changes in the left thalamus of MDDs, negatively correlated with depression severity measured by HAMD. Additionally, the reduced fractional anisotropy in the anterior thalamic nucleus, vital for emotional regulation, underscores its diagnostic and therapeutic potential [63]. We hypothesize that these changes could be linked to neurotransmitter abnormalities or alterations in neuronal connectivity, potentially affecting thalamic coupling. Aligned with our findings, studies have shown altered thalamic FC in MDD with anterior cingulate cortex [52], temporal, somatosensory areas [64,65], superior frontal gyrus [60], and anterior prefrontal cortex [66], among others. Furthermore, some of the alterations were related to severity of symptoms and neuropsychological functioning of patients. For instance, thalamo-temporal connectivity positively correlated with symptom severity [64]. Higher ruminative response scale and repetitive negative thinking scores in MDDs were associated with increased connectivity of the right thalamus to specific brain regions [67]. Additionally, the variance of dynamic FC between the left sensory thalamus and the right inferior temporal gurus/fusiform correlated positively with the childhood trauma scores [68], while somatosensory-thalamic FC had a negative correlation with the assessment of neuropsychological status total score, delayed memory score, and two-digit continuous operation test score in MDDs [65]. Consequently, the central role of the thalamus in integrating and regulating emotional information may be compromised. Such instability and functional inhibition can lead to a decline in emotional responses and cognitive abilities, further impacting the patient’s daily life and mental health.

### Potential biomarker and related previous work for ASDs

Ou analysis of bifurcation parameters of ASDs showed that R Calcarine, R Posterior Cingulate Gyrus(PCC), R Cuneus, R Median Gingulate and Paracingulate Gyrus(MCC), R Insula, L Posterior Cingulate Gyrus(PCC), R Anterior Cingulate, and Paracingulate Gyri (ACC) were statistically significant and also showed prominent effect sizes. Moreover, these areas had considerable weights in the linear-SVM classifier, which could be potential biomarkers to distinguish between HCs and ASDs. These brain regions are closely associated with perception, emotion, cognitive, and social functions. Compared to HCs, we found that bifurcation parameters of these brain regions of ASDs tended to be closer to zero. This indicated a reduction in intrinsic activation levels and driving forces, which may have led to a decrease in spontaneous activity, wich was also reported previously that reduced activity in the ACC and insular is linked to diminished attention and control in ASDs [69,70]. As an essential region for emotion regulation, attention control, decision-making, social behavior, and pain perception [71,72], the Cingulate cortex is highlighted by Chiu et al. [73] as having a reduced self-response, a neural marker in high-functioning autism. Complementary findings indicate that the MCC’s activation during social decisions and emotions emphasizes its role in ASDs [74], and neuronal impairments are evident from cellular and metabolic differences in the ACC and PCC [75]. Our findings align with those of Leech and Sharp [76], where low activity in the dorsal PCC is linked to decreased metastability, potentially leading to cognitive inflexibility, a hallmark feature of ASD. Additionally, we have validated that as the parameter approached the critical point, FC between this region and others showed unpredictability and potential non-monotonic behavior. We hypothesize that such variability could significantly influence the function of the Insula either, which is a pivotal hub for integrating sensory information, emotions, self-awareness, and cognitive processes [77]. Supporting our hypothesis, studies have linked insular dysfunction to social impairments through abnormalities in resting-state connectivity [70,78] and identified partial impairments in its connectivity with the default mode network [41,79]. Further investigations have revealed variations in cortical thickness and global connectivity of the insular cortex between ASDs and HCs, underscoring its significant role in neural integration [80,81]. These findings collectively illustrate the multifaceted role of these regions in ASD, significantly impacting the behavioral and functional performance of individuals with ASD.

In addition, we found that the parameter values in the right SPG of ASDs are significantly higher than those in HCs. However, there is a significant negative correlation between the parameter values of the SPG and the severity of social impairment (ADOS-Social scores). Greene et al. [82] has provided a strong explanation and support for our findings that in typically developing (TD) group, the frontoparietal attention network, visual processing areas, and the striatum exhibited significantly enhanced activity in response to social cues compared to non-social cues. In contrast, the ASD group exhibited increased activity only in the SPG, without significant differences. This suggests that the neural circuits for social orientation in ASD are impaired, prompting individuals to adopt compensatory mechanisms for processing social information, rather than utilizing a fully functional social attention system. Based on this, we speculate that as a compensatory brain region, the activation level of the SPG in ASDs is higher than in HCs. However, as social skills deteriorate (indicated by higher ADOS-Social scores), this compensatory ability decreases. Apart from this trend, it is widely held that the SPG is a critical region for processing complex visual inputs and understanding spatial relationships, essential for interpreting human behavior and managing social interactions, and also maintains connections with key areas such as the ACC and the insula, which are integral to emotional and social functioning. Consequently, we propose that abnormalities in SPG activity or connectivity may contribute to deficits in sophisticated cognitive functions and social communication skills in ASD, particularly impairing the interpretation of body language and spatial cues. Supporting this proposition, numerous studies have demonstrated that reduced functional connectivity in the SPG of ASDs has been shown to affect self-perception and theory of mind [83,84], and significant structural changes, connectivity issues, and decreased activity during spatial attention and visuomotor tasks in the parietal lobes of ASDs were reported [85,86].

### Classification accuracy for MDDs and ASDs

A review of classification studies on MDD and ASD datasets reveals that although research on smaller datasets may report higher classification accuracies, extensive neuroimaging research indicates that predictive accuracy actually decreases with an increase in sample size and clinical heterogeneity [87,88]. However, this improves the model’s generalizability and ensures the reliability of biomarker inference [89]. Based on these concepts, our work utilized the largest MDD and ASD resting-state fMRI datasets, applying bifurcation parameters as features in basic SVM classifiers. Compared to studies using the same datasets and sample size that employed functional connectivity features, our approach not only demonstrated superior performance with the same classifier (Tables 1 and 2) but also surpassed many studies using more complex models such as graph convolutional networks (GCNs) and deep neural networks (DNNs). Although GCN is specifically designed to capture topological data characteristics, and DNN exhibits strong predictive capabilities in image and sequence processing, our straightforward SVM method based on bifurcation parameters still significantly excelled in accuracy, as shown in Tables S3 and S4. This notable performance advantage revealed several key insights: Bifurcation parameters sensitively and specifically capture the underlying dynamics of various brain regions from time-series data; focusing on low-dimensional yet informative features reduces model complexity and overfitting risks, enhancing generalizability; moreover, this method can intuitively depict the underlying mechanisms of brain dynamics, featuring strong interpretability and operability, with promising prospects for clinical application. These findings challenge the conventional belief that more complex models invariably yield better outcomes, highlighting the significant potential of dynamical systems theory in enhancing the accuracy of neuroimaging diagnostics and predictions.

### Conclusion and future perspectives

In summary, the adaptive parameter fitting method for the Hopf whole-brain model inspired by the synthetic model has demonstrated exceptional predictive accuracy with resting-state fMRI BOLD signals in MDD and ASD datasets, showcasing its potential as a versatile and robust brain state fitting tool. Compared to existing methods, it offers significant advantages in adaptability, predictive accuracy, and computational efficiency, making it especially suitable for analyzing signals from complex and diverse brain parcellations. This flexibility and reliability establish the method’s broad relevance to the study of various mental disorders and conditions, providing critical tools and evidence not only for neuroscience research but also for clinical diagnosis and treatment planning.

Looking ahead, our synthetic model-inspired adaptive parameter fitting method can be extended to other neuroimaging techniques like MEG, EEG, and near-infrared spectroscopy (NIRS), providing comprehensive neural state fitting across different fields. Moreover, combining this approach with multimodal physiological data will help enhance its predictive and classification abilities. By leveraging transfer learning, we can apply this framework to different synthetic models or neural network structures to optimize their performance in various fields. Further development will enable its use in real-time monitoring and feedback systems, making it applicable for real-time brain–computer interfaces or clinical monitoring. Additionally, this method can be extended to Alzheimer’s, Parkinson’s, epilepsy, and other brain disorders to verify its broad adaptability. Ultimately, by identifying individualized brain state patterns, it will be able to predict disease progression early and tailor treatment strategies, providing precise therapeutic recommendations for patients.

## Materials and Methods

### Model

The local dynamics of each node are described by the standard form of a supercritical Hopf bifurcation, which models the transition from asynchronous noisy behavior to full oscillations:

$$\frac{\mathrm{dz}}{\mathrm{dt}}=\left(a+\mathrm{i\omega}-{\left|z\right|}^{2}\right)z+\beta \eta \left(t\right)$$ (4)

Here, z=x+iy, where x and y represent the real and imaginary parts of the state variable z, respectively (in arbitrary units), and i is the imaginary unit. The modulus of z is given by $\left|z\right|$ such that ${\left|z\right|}^{2}=x^{2}+y^{2}$. The Hopf whole-brain model consists of N coupled brain regions (ROIs), where the number of nodes depends on the selected brain parcellation atlas. The global dynamics of the model emerge from the interaction between local node dynamics and the empirical SC matrix $C_{\mathrm{ij}}$, derived from DTI-based tractography. After separating the real and imaginary parts of the complex equation and incorporating coupling effects, the whole-brain dynamic equation is defined as:

$$\frac{dx_{i}}{dt}=[a_{i}-x_{i}^{2}-y_{i}^{2}]x_{i}-\omega_{i}y_{i}+G\sum_{j}C_{ij}(x_{j}-x_{i})+\beta \eta_{i}(t)$$ (5)

$$\frac{dy_{i}}{dt}=[a_{i}-x_{i}^{2}-y_{i}^{2}]y_{i}+\omega_{i}x_{i}+G\sum_{j}C_{ij}\left(y_{j}-y_{i}\right)+\beta \eta_{i}(t)$$ (6)

Here, the SC matrix $C_{\mathrm{ij}}$ represents the fiber density between cortical regions i and j, with all matrix values normalized to a maximum of 0.3. The variable $x_{n}$ emulates the BOLD signal of each ROI n. In this model, the parameter $a_{i}$ is known as the bifurcation parameter and controls the dynamical behavior of the system; *G* denotes the global coupling weight, scaling SC equally for each brain area; $\eta_{i}\left(t\right)$ is additive Gaussian noise with standard deviation = 0.02; the intrinsic frequency $\omega_{i}$ of each ROI is in the 0.01 to 0.1 Hz band $\left(i=1,\;\ldots ,\;n\right)$ and is fitted from the data, as given by the averaged peak frequency of the narrowband BOLD signals of each brain region: $f_{i}=\omega_{i}/2\pi$.

Within this model, 2 parameters are needed to be optimized: The global scaling factor *G* and the bifurcation parameters $a_{i}$. These parameters act as control variables, enabling us to investigate the ideal dynamic working region where the simulation closely aligns with the empirical FC and FCD of BOLD signals.

### Simulation and validation

To validate the wide applicability of the fitting method, we conducted tests on various simulated networks that reflect the characteristics of the biological brain. Initially, in the simulated networks, the connection density reached 35%, a value that has been confirmed in numerous studies. When constructing the network, we drew connection weights from the exponential distribution. To enhance the diversity and randomness of the network, we adopted 2 distinct network generation strategies: the Watts–Strogatz (WS) model and the Barabási–Albert (BA) model. The WS model takes into account the probability P of rewiring. In this context, we experimented with multiple values of P, such as P=0, P=0.35, P=0.5, and P=1, introducing randomness by disconnecting network edges and reconnecting them to randomly selected vertices. Meanwhile, in the BA model, with a connection density of P=0.35 as the benchmark, we simulated a scale-free network characteristic, where certain nodes possessed numerous connections while others had only a few. Ultimately, these simulated network structures will be used for further validation of the fitting method. This will help us be confident that the proposed method performs well in a variety of complex networks. A schematic of the simulated network structure can be seen in Fig. 1B.

### Functional connectivity dynamics

In this study, we used FCD to detect recurring network state patterns by analyzing phase-interaction pattern repetition. This method builds on prior work defining FCD for FC matrices over different time windows.To calculate the FCD, the scan duration was divided into sliding windows, each containing 30 time points, with a time repetition (TR) shift between consecutive windows. For each window centered at time , we computed the average phase-interaction matrix $\langle P\left(t\right)\rangle$ as:

$$\langle P\left(t\right)\rangle =\frac{1}{T}\sum_{t_{0}\in \text{window}}P\left(t_{0}\right)$$ (7)

where T is the total number of TRs and $P(t_{0})$ is the phase-interaction matrix at time $t_{0}$. Next, an M×M symmetric matrix was constructed, with each entry $\left(t_{1},t_{2}\right)$ defined by the cosine similarity $S_{\cos}$ between the vectorized forms of $\langle P(t_{1})\rangle$ and $\langle P(t_{2})\rangle$:

$$S_{\text{cos}}\left(t_{1},\;t_{2}\right)=\frac{p\left(t_{1}\right)\cdot p\left(t_{2}\right)}{∥p\left(t_{1}\right)∥∥p\left(t_{2}\right)∥}$$ (8)

where $p(t_{1})$ and $p(t_{2})$ are the vectorized forms of the phase-interaction matrices, and θ is the angle between the vectors. The cosine of θ quantifies the similarity between phase-interaction patterns at times $t_{1}$ and $t_{2}$. Finally, the FCD measure is represented by the distribution of cosine similarities across all time-window pairs, capturing the dynamics of functional connectivity over time.

### Data

#### REST-meta-MDD data

In the REST-meta-MDD Project of the DIRECT consortium [36,37], 25 research groups from 17 hospitals in China agreed to share final R-fMRI indices from patients with MDD and matched normal controls from studies approved by local Institutional Review Boards. The consortium shared a total of 2,428 datasets, including 1,300 MDDs and 1,128 HCs. We excluded subjects who did not meet the age criteria (18 to 65) and had incomplete information, low image quality (by visual inspection), bad spatial normalization, bad coverage (<0.9), short sampling time (time steps < 190), low depression score (HAMD < 7), inappropriate TR, excessive head movement (mean framewise displacement > 0.2 mm), and abnormal fALFF indices. After this filtering process, we selected 666 MDDs and 648 HCs for in-depth parameter fitting and statistical analyses. Among these MDD patients, females were in the majority, totaling 427 cases, compared to 239 male cases. There were 138 recurrent MDDs and 317 first-episode MDDs (159 FEDN and 100 FEMed MDDs). Regarding medication status, there are 207 MDDs not on medication and 179 MDDs on medication. The detailed exclusion and inclusion process is illustrated in Fig. S5. The composition of the MDD subjects is shown in Table 3. In addition, the only shared information are the following: subject ID, sex, age, education, episode status, medication status, illness duration, HAMD, and Hamilton anxiety rating scale (HAMA).

**Table 3.** Composition of MDD participants

Group	NUM	Group	NUM
MDD non-Medicated	207	ASD non-Medicated	184
MDD Medicated	179	ASD Medicated	67
MDD FEDN	159	ASD Hand-Left	25
MDD FEMed	100	ASD Hand-Right	183
MDD Female	427	ASD Female	41
MDD Male	239	ASD Male	292
MDD First Episode	317		
MDD Recurrent	138		

In this study, we utilized node BOLD signal data from the dataset that underwent global regression processing. This data preprocessing was carried out by the direct alliance of the REST-meta-MDD project (refer to Yan et al. [90] and Chen et al. [37]). Each participating local site adopted a standardized preprocessing pipeline based on the resting-state fMRI data processing assistant (DPARSF) [91] to minimize the heterogeneity between preprocessing methods. In brief, this procedure includes removing the first 10 volumes for signal stabilization, slice timing correction, head motion realignment, brain tissue segmentation, spatial normalization, and temporal frequency filtering (0.01to0.10 Hz). Moreover, ALFF and fALFF were extracted from the shared dataset, using the DPARSF.

#### ABIDE data

ABIDE-I, a multi-site platform, aggregates functional and structural brain imaging data collected from 16 different laboratories globally. The original Preprocessed Connectomes Project (PCP) of ABIDE has publicly released data on 539 individuals with ASD and 573 HC individuals [41,42]. This dataset of 1,112 subjects comprises structural and preprocessed resting-state functional MRI (rs-fMRI) data along with phenotypic information. The rs-fMRI data underwent slice-time correction, motion correction, and normalization. There are 4 preprocessing pipelines including CCS, CPAC, DPARSF, and NIAK for the dataset. To maintain consistency with the REST-meta-MDD dataset, only the DPARSF preprocessing data were used in our analysis and subjected to band-pass filtering (0.01to0.1Hz). From these subjects, 746 were selected, meeting the assessment criteria, possessing complete phenotypic information, and fitting our model requirements for our study. Among these 746 subjects, there were 333 with ASD and 413 HC, including 122 females and 624 males. A summary of the selected ASD subjects is presented in Table 3. Detailed information about the public data of ABIDE-I is available online (http://fcon_1000.projects.nitrc.org/indi/abide/abide_I.html).

#### Structural connectivity

To ascertain SC, we utilized data shared by Škoch et al. [92]. This dataset furnishes SC matrices of 88 subjects. The construction of the SC matrices was based on a connectome generated by probabilistic tractography of diffusion MRI data and employs ROIs from the widely used Automated Anatomical Labeling atlas (AAL) [93], version ROI_MNI_v4 to delineate the connectivity matrices. Comprehensive details on the data acquisition and preprocessing procedures are elaborated within the article.

Maier-Hein et al. [94] demonstrated that existing tractography pipelines, even when tracking the ground truth fiber orientations on high-resolution images, generate a considerable amount of false-positive bundles. It is noteworthy that probabilistic tractography imaging yields an almost fully connected weighted network, inundated with a significant number of incidental fibers. To enhance the reliability of connection fitting, it becomes imperative to trim these erroneous connections stemming from the probabilistically generated network [95]. In response to this challenge, we implemented 2 methodical network thresholding strategies: consistency and proportional thresholding.

The consistency-thresholding method retains connections with weights that remain uniform across subjects. This is grounded in the assumption that connections exhibiting the most considerable intersubject variability are likely to be spurious. Our approach complements the consensus-based thresholding traditionally applied to networks derived from deterministic tractography. Specifically, we selected edges that appeared in a minimum of 75% of subjects and subsequently averaged these edges across the 88 subjects [95]. Subsequent to this, we employed the proportional thresholding technique, which sets a relative threshold on the connection weights. This ensures the removal of the weakest connections while simultaneously preserving 35% of the strongest links [94,96].

It should be noted that in this shared dataset, the SC matrices obtained by the standard methods are not symmetrical. Since the interpretation of the direction of these links is not straightforward, the matrices were symmetrized by calculating ${\mathrm{SC}}_{\mathrm{sym}}=(\mathrm{SC}+SC^{\prime })/2$ before being used.

### Classification methods

During the classification process using 5-fold cross-validation, we performed a preliminary analysis to check the distribution of the data, and when necessary (class imbalance), we applied oversampling method for the minority class to ensure a balanced representation in the training set. After that, we chose the default parameter C=1 for linear-SVM. For rbf-SVM, we first divided the dataset into training and testing parts. Subsequently, we employed a 5-fold stratified cross-validation method to ensure that the category distribution in each fold was roughly the same. Within rbf-SVM, C and γ are the 2 core parameters of the RBF kernel. Specifically, C is the regularization factor of SVM, also referred to as the penalty coefficient, balancing the trade-off between the shape of the decision boundary and misclassification. A smaller C value results in a wider boundary but may allow some misclassifications. Conversely, a larger C value tends to produce a narrower margin, even if this leads to more data points crossing that margin. Gamma determines the shape of the decision boundary, essentially defining the influence range of each sample. A smaller γ means that each sample has a broader influence range, while a larger γ could result in a more intricate decision boundary. To find the optimal parameters for SVM, we set up a parameter search grid, with candidate values for C being $\left[1,10,100,1000\right]$ and for γ being $\left[0.0001,0.001,0.01,0.1\right]$. Upon identifying the best parameters, we retrained the SVM on the full training data using these parameters and assessed its performance on the test set. Besides balanced accuracy, we also computed other performance metrics like accuracy, precision, recall, and F1 score, and recorded their mean values and standard deviations across the 5-fold cross-validation. All hyperparameter tuning and SVM classifications were performed using the Scikit-Learn Python machine learning package [97].

Balanced accuracy, accuracy, precision, recall, and F1 score are critical indicators for evaluating the performance of classification models. Their definitions and calculations are as follows:

$$\text{Accuracy}=\frac{\mathrm{TP}+\mathrm{TN}}{\mathrm{TP}+\mathrm{TN}+\mathrm{FP}+\mathrm{FN}}$$ (9)

$$\text{Precision}=\frac{\text{TP}}{\text{TP}+\text{FP}}$$ (10)

$$\text{Recall}=\frac{\text{TP}}{\text{TP}+\mathrm{FN}}$$ (11)

$$F1=2\times \frac{\text{Precision}\times \text{Recall}}{\text{Precision}+\text{Recall}}=\frac{2\text{TP}}{2\text{TP}+\text{FP}+\mathrm{FN}}$$ (12)

where TP is true positives, TN is true negatives, FP is false positives, and FN is false negatives.

### Statistical analyses

#### Linear mixed models

LMM, also known as a hierarchical linear models or mixed-effects models, is an extension of the simple linear regression model that can simultaneously account for fixed and random effects. We used LMM to compare MDDs and ASDs with HCs, allowing for site variation effects in the model. We utilized MATLAB’s “fitlme” command for model fitting (see: https://www.mathworks.com/help/stats/fitlme.html). In the MDD model, we set each individual’s HAMD score as the dependent variable, each ROI’s bifurcation parameter $a_{i}$ as fixed effects, and the intercepts influenced by site differences as a random effect. Age, gender, educational level, and head motion were included as covariates to control for potential confounders: $\begin{array}{c} \begin{array}{c} y∼1+a_{i}+\text{Age}+\text{Sex}+\text{Education}+\text{Motion}+\left(1|\;\text{Site}\right) \end{array} \end{array}$, which generates t values and *P* values for the fixed effects of $a_{i}$. For the ASD model, each individual’s ADOS-Social score was set as the dependent variable, with each ROI’s bifurcation parameter $a_{i}$ as fixed effects. Age and gender were used as covariates to control for potential confounding factors: $y∼1+a_{i}+\text{Age}+\text{Sex}+\left(1|\;\text{Site}\right)$, also generating t values and P values for the fixed effect of $a_{i}$. The FDR was used to correct for multiple comparisons across ROIs, setting a *P* value threshold of <0.05 for corrections.

#### *t* Test

The *t* test is a statistical method used to compare whether there is a significant difference in the means of 2 groups of data. It can be applied to 2 independent samples, paired samples, or comparing a single sample with a known value. Through the *t* test, we can obtain a *t* statistic and the corresponding *P* value to determine the statistical significance of the difference. If the *P* value after FDR correction is lower than a predetermined significance level (0.05), the difference is considered statistically significant. However, this does not directly indicate the actual size or meaning of the difference, so the effect size is often calculated for reference.

#### Effect size

Cohen’s *d* is an effect size metric used to represent the standardized difference between the means of 2 groups. Its formula is:

$$d=\frac{M_{1}-M_{2}}{s},$$ (13)

where $M_{1}$ and $M_{2}$ are the means of the 2 groups being compared, and s is the standard deviation, typically the pooled standard deviation of the 2 groups. The value of Cohen’s *d* describes the magnitude of the difference between 2 groups: d<0.2 is typically considered a “small” effect size, 0.2<d<0.5 is considered a “medium” effect size, and d>0.8 is viewed as a “large” effect size. Cohen’s *d* is particularly useful in reporting research results, meta-analyses, and power analyses, as it offers a measure of the difference’s size independent of the sample size.

## Data Availability

All data used in the paper are present in the paper and/or the Supplementary Materials. The MATLAB code for the whole-brain models and fitting procedures is available on GitHub (https://github.com/B-and-ILab/Adaptive-Whole-Brain-Dynamics-Predictive-Method-Relevancy-to-Mental-Disorders.git).

## Supplementary Materials

Supplementary 1 Figs. S1 to S5 Tables S1 to S4 Appendix References
