# Supplementary material for: Adaptive Whole-Brain Dynamics Predictive Method: Relevancy to Mental Disorders
Source: Research (Wash D C). 2025 Apr 5;8:0648. doi: 10.34133/research.0648 (PMC11971527; doi:10.34133/research.0648)

Simulated Powers 264 functional ROIs (Power et al., 2011)

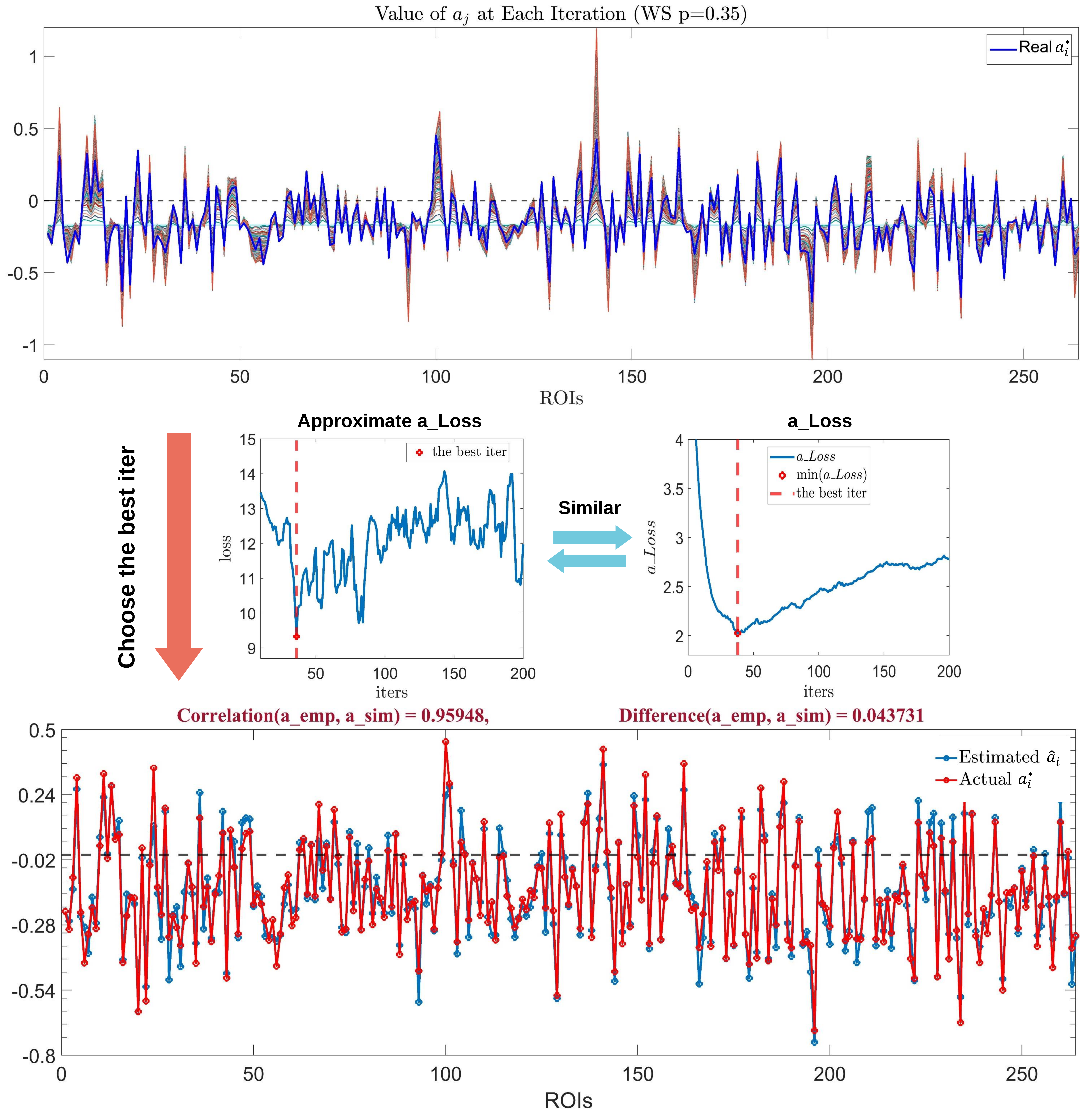

Supplement: Supplementary 1 — Figs. S1 to S5 Tables S1 to S4 Appendix References [file research.0648.f1.zip › FigS1.pdf]
