# Supplementary material for: Adaptive Whole-Brain Dynamics Predictive Method: Relevancy to Mental Disorders
Source: Research (Wash D C). 2025 Apr 5;8:0648. doi: 10.34133/research.0648 (PMC11971527; doi:10.34133/research.0648)
Supplement: Supplementary 1 — Figs. S1 to S5 Tables S1 to S4 Appendix References [file research.0648.f1.zip › FigS2.pdf]

(a)

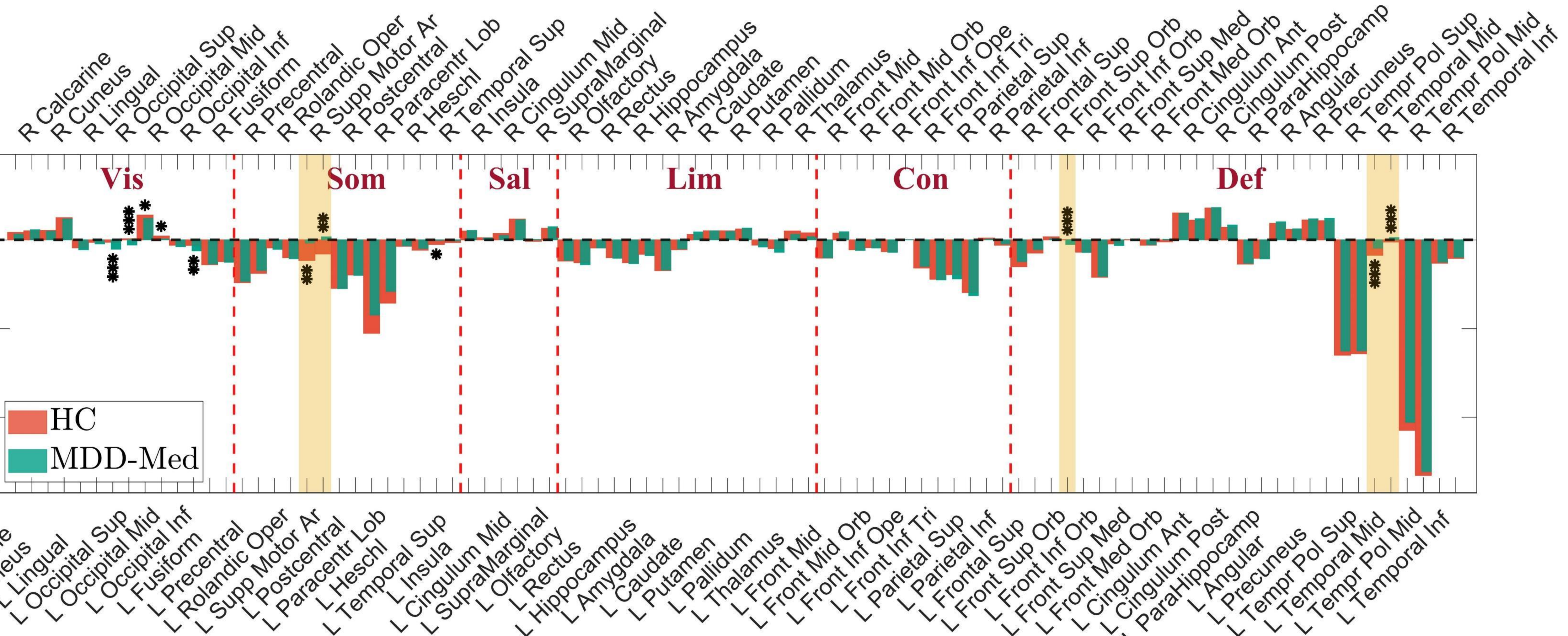

ROIs	Linear-SVM (ACC: 83.81%)	t-test	
	Weight	p	Cohens d
L Temporal Mid	3.6960	$< 0.0001^{***}$	−0.7378
R Supp Motor Ar	2.5668	0.0016^{**}	−0.4384
R Front Sup Orb	−2.3687	0.0010^{**}	0.4614
R Temporal Mid	2.3450	$< 0.0001^{***}$	−0.5980
L Supp Motor Ar	1.8342	0.0017^{**}	−0.4328

(b)

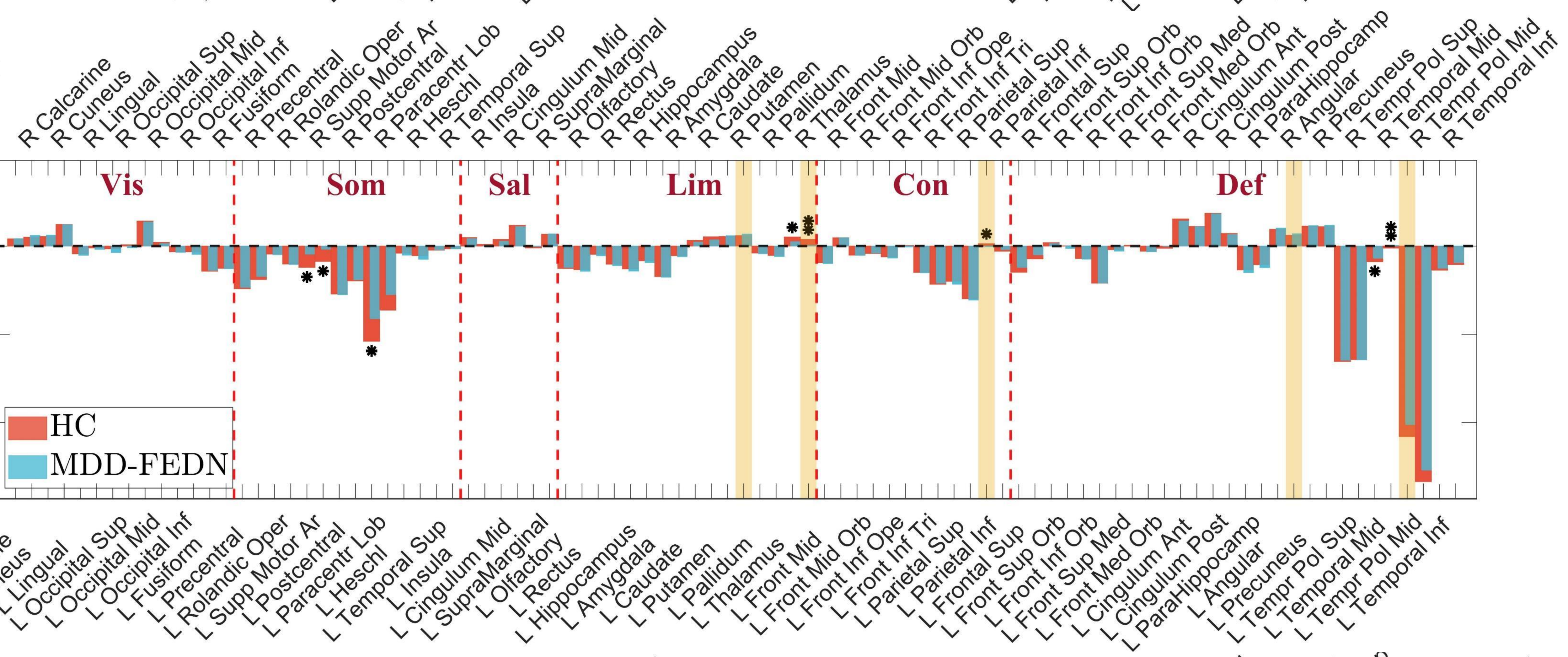

ROIs	Linear-SVM (ACC: 74.33%)	t-test	
	Weight	p	Cohens d
R Angular	2.4063	0.1616	−0.2030
L Parietal Inf	−2.1919	0.0128^{*}	0.3266
R Thalamus	−2.1227	0.0017^{**}	0.4169
R Putamen	1.9184	0.4260	−0.1167
L Tempr Pol Mid	1.7441	0.2218	−0.1825

(c)

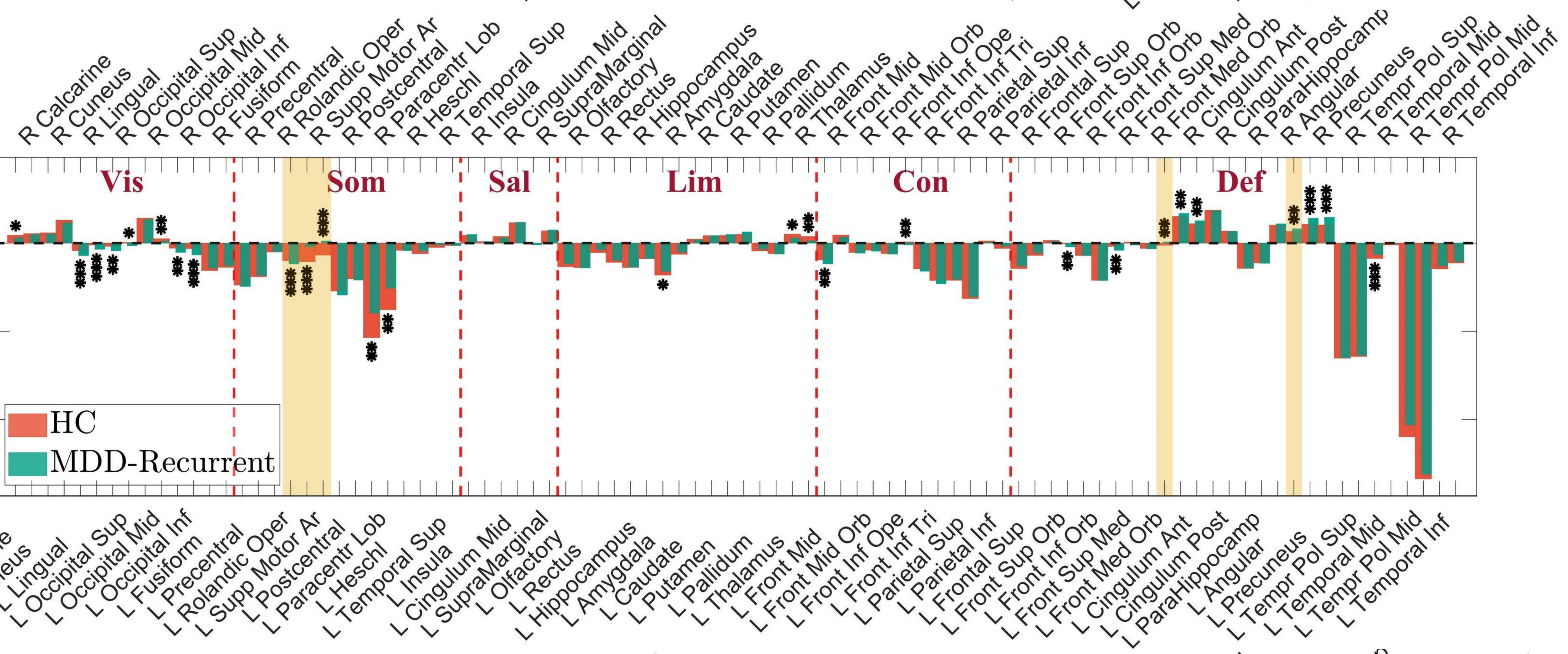

ROIs	Linear-SVM (ACC: 81.75%)	t-test	
	Weight	p	Cohens d
R Angular	2.9172	0.0050^{**}	−0.3245
R Rolandic Oper	−2.6340	0.0004^{***}	0.4165
R Supp Motor Ar	2.2418	0.0007^{***}	−0.3921
R Front Med Orb	2.1749	0.0077^{**}	−0.3034
L Supp Motor Ar	2.0127	0.0004^{***}	−0.4147

(d)

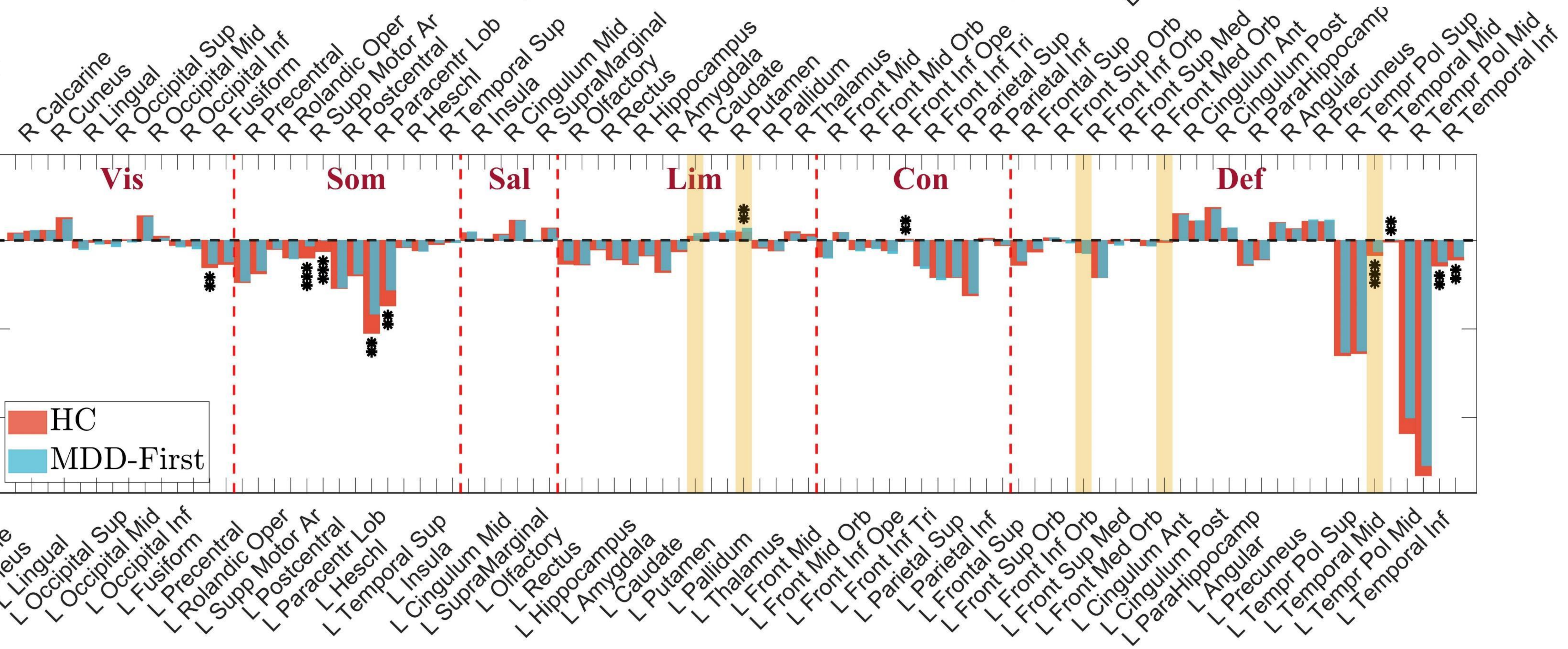

ROIs	Linear-SVM (ACC: 70.66%)	t-test	
	Weight	p	Cohens d
R Front Med Orb	2.7884	0.0848	−0.1590
L Temporal Mid	2.1134	0.0001^{***}	−0.3424
L Caudate	2.0234	0.0221^{*}	−0.2095
R Putamen	1.8667	0.0025^{**}	−0.2680
L Front Inf Orb	−1.8518	0.1579	0.1328
