# Supplementary material for: Adaptive Whole-Brain Dynamics Predictive Method: Relevancy to Mental Disorders
Source: Research (Wash D C). 2025 Apr 5;8:0648. doi: 10.34133/research.0648 (PMC11971527; doi:10.34133/research.0648)
Supplement: Supplementary 1 — Figs. S1 to S5 Tables S1 to S4 Appendix References [file research.0648.f1.zip › FigS3.pdf]

(a)

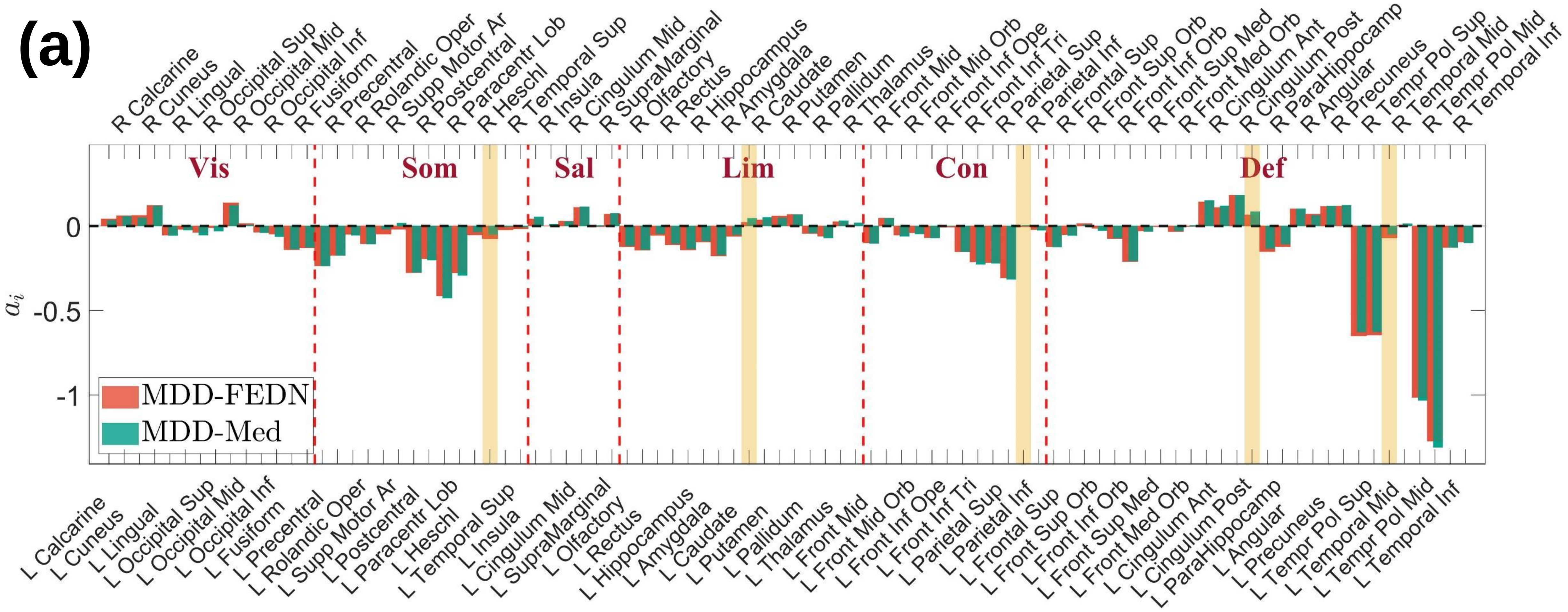

ROIs	Linear-SVM (ACC: 60.06%)	t-test	
	Weight	p	Cohens d
L Temporal Mid	−1.9909	0.2790	0.3548
L Caudate	−1.9797	0.2790	0.3522
L Parietal Inf	−1.9030	0.8310	0.1338
R Heschl	−1.4227	0.6896	0.2446
R Cingulum Post	−1.3616	0.6896	0.1814

(b)

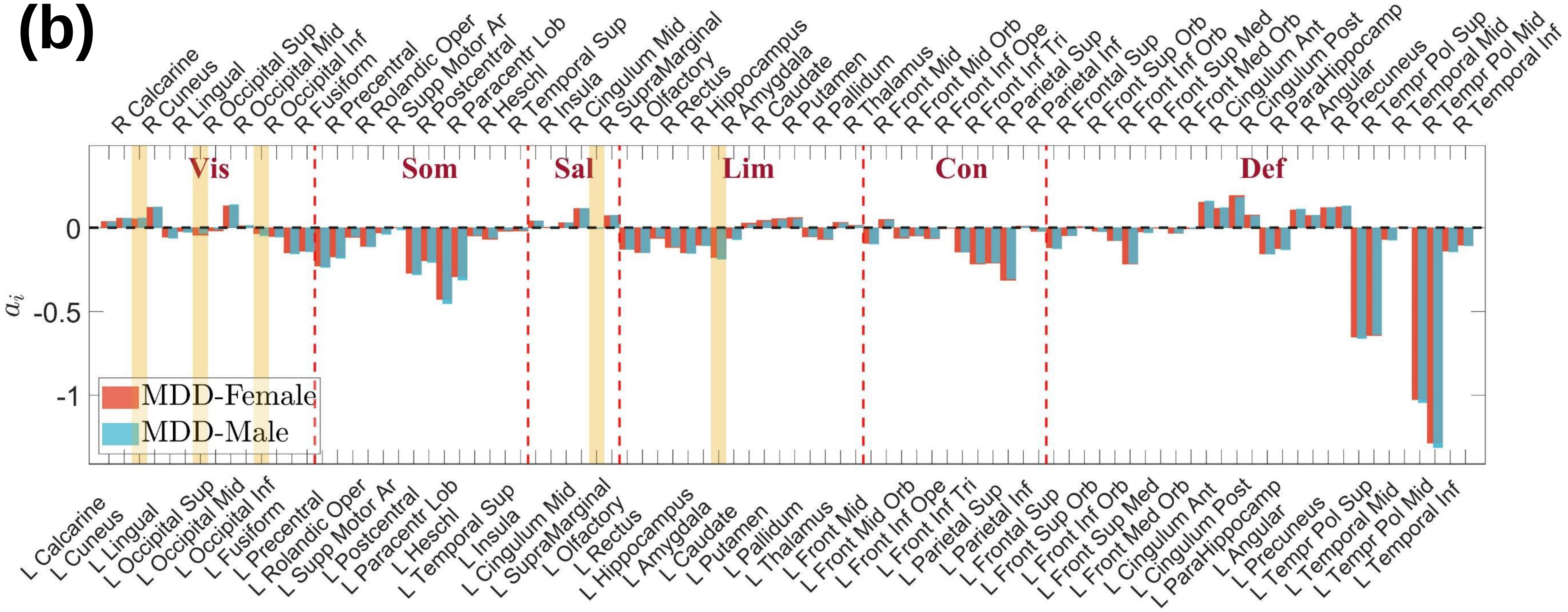

ROIs	Linear-SVM (ACC: 58.20%)	t-test	
	Weight	p	Cohens d
L Occipital Inf	2.4864	0.8312	−0.1812
L Cuneus	−1.8537	0.8312	0.1078
L SupraMarginal	1.7556	0.8312	−0.0905
L Amygdala	1.7544	0.8312	−0.1381
L Occipital Sup	−1.7378	0.8312	0.1173

(c)

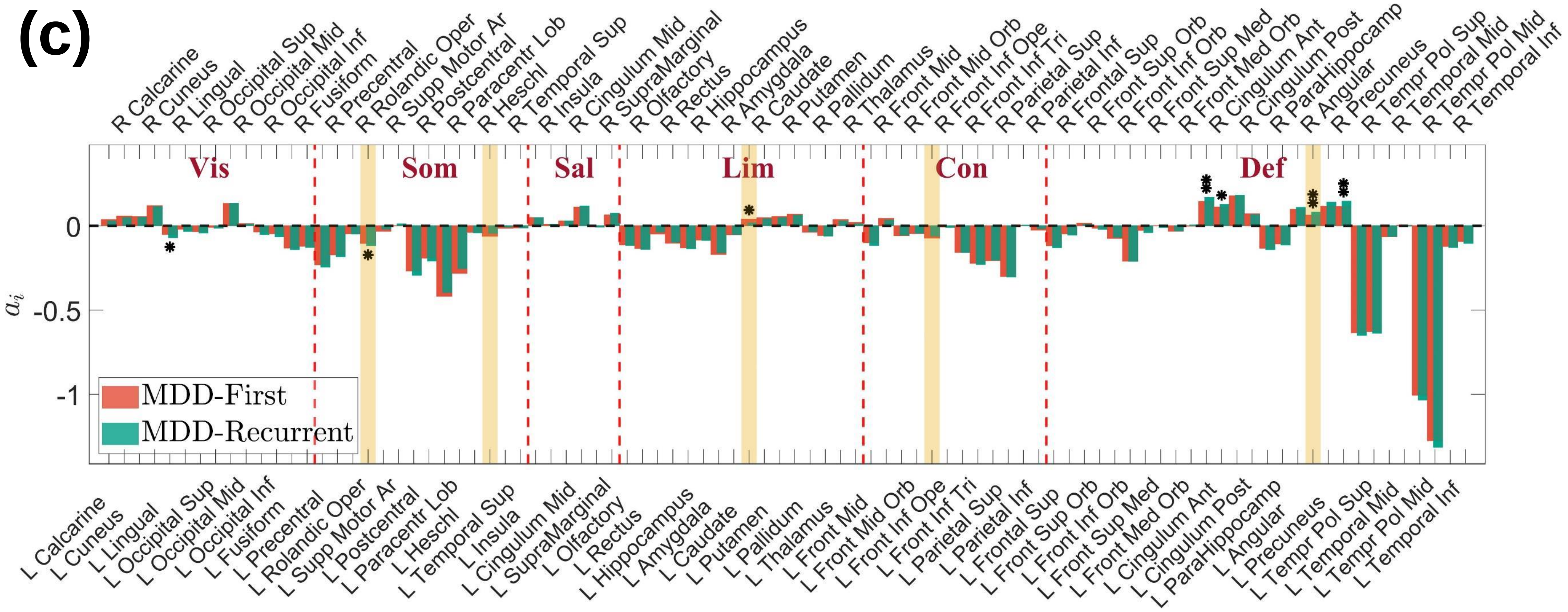

ROIs	Linear-SVM (ACC: 72.09%)	t-test	
	Weight	p	Cohens d
R Heschl	2.5632	0.3208	0.1826
R Angular	−2.4087	0.0040^{**}	0.3968
R Rolandic Oper	−2.0196	0.0180^{*}	−0.3377
L Front Inf Ope	1.8084	0.1937	0.2114
L Caudate	−1.7621	0.0213^{*}	−0.3274
