# Supplementary material for: Adaptive Whole-Brain Dynamics Predictive Method: Relevancy to Mental Disorders
Source: Research (Wash D C). 2025 Apr 5;8:0648. doi: 10.34133/research.0648 (PMC11971527; doi:10.34133/research.0648)
Supplement: Supplementary 1 — Figs. S1 to S5 Tables S1 to S4 Appendix References [file research.0648.f1.zip › FigS4.pdf]

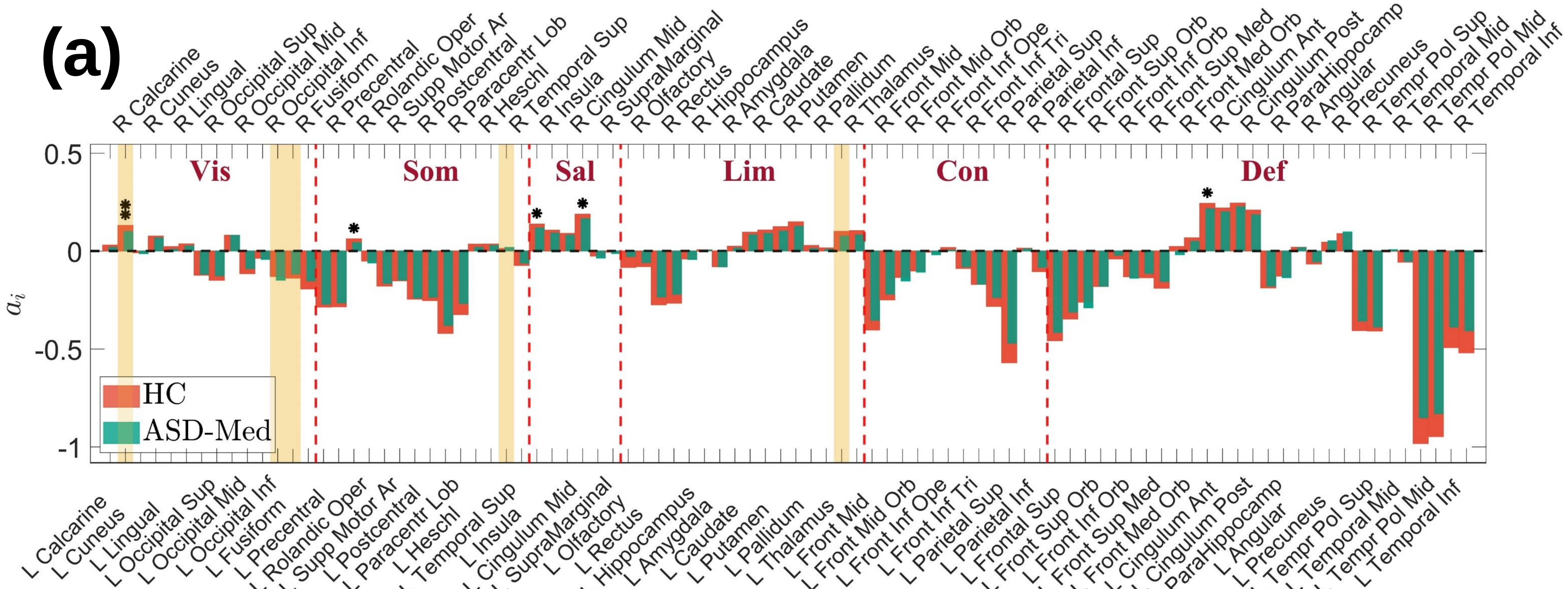

ROIs	Linear-SVM (ACC: 82.88%)	t-test	
	Weight	p	Cohens d
R Calcarine	−3.6806	0.0026**	0.5723
R Occipital Inf	−2.0814	0.5790	0.1324
L Temporal Sup	2.0606	0.6495	−0.0933
L Thalamus	−1.6665	0.0606	0.3786
L Fusiform	1.6511	0.2420	−0.2559

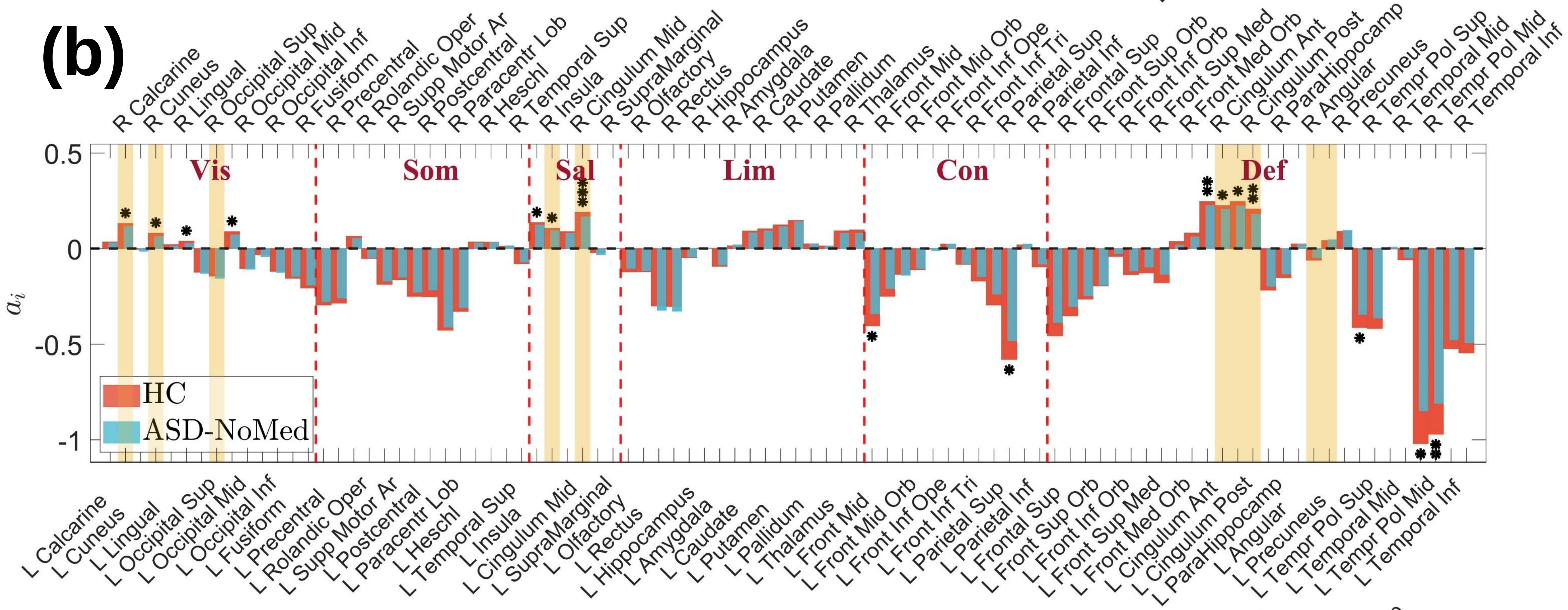

ROIs	Linear-SVM (ACC: 76.11%)	t-test	
	Weight	p	Cohens d
R Occipital Sup	−2.6801	0.5846	0.0823
R Calcarine	−2.1373	0.0295*	0.2873
R Cingulum Post	−2.0344	0.0196*	0.2997
R Cingulum Mid	−1.9130	0.0003***	0.4345
L Precuneus	−1.7917	0.7636	−0.0445
R Angular	1.7122	0.2671	−0.1411
R Cingulum Post	−1.6712	0.0101*	0.3241
R Cuneus	−1.5913	0.0359*	0.2575
R Cingulum Ant	−1.3539	0.0196*	0.2965
R Insula	−1.4634	0.0250*	0.2771

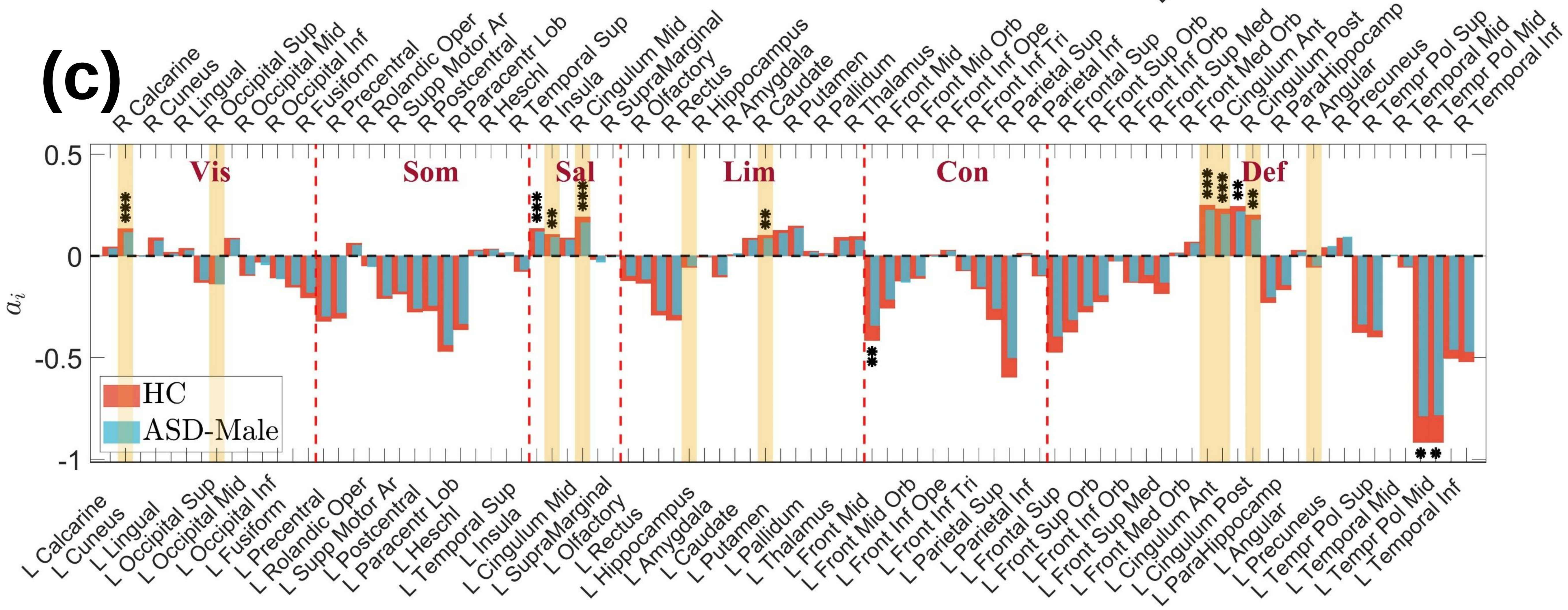

ROIs	Linear-SVM (ACC: 71.54%)	t-test	
	Weight	p	Cohens d
R Occipital Sup	−2.4017	0.9461	0.0054
R Calcarine	−1.9935	0.0006**	0.3365
R Cingulum Post	−1.7965	0.0057**	0.2958
L Hippocampus	1.7929	0.3148	−0.1118
R Cingulum Mid	−1.7562	< 0.0001***	0.4867
R Caudate	−1.7451	0.0064**	0.2711
R Insula	−1.6836	0.0012**	0.3133
L Cingulum Ant	−1.4663	< 0.0001***	0.4109
R Cingulum Ant	−1.2579	0.0001***	0.3854
R Angular	1.2312	0.4809	−0.0807
