# Supplementary material for: Adaptive Whole-Brain Dynamics Predictive Method: Relevancy to Mental Disorders
Source: Research (Wash D C). 2025 Apr 5;8:0648. doi: 10.34133/research.0648 (PMC11971527; doi:10.34133/research.0648)
Supplement: Supplementary 1 — Figs. S1 to S5 Tables S1 to S4 Appendix References [file research.0648.f1.zip › FigS5.pdf]

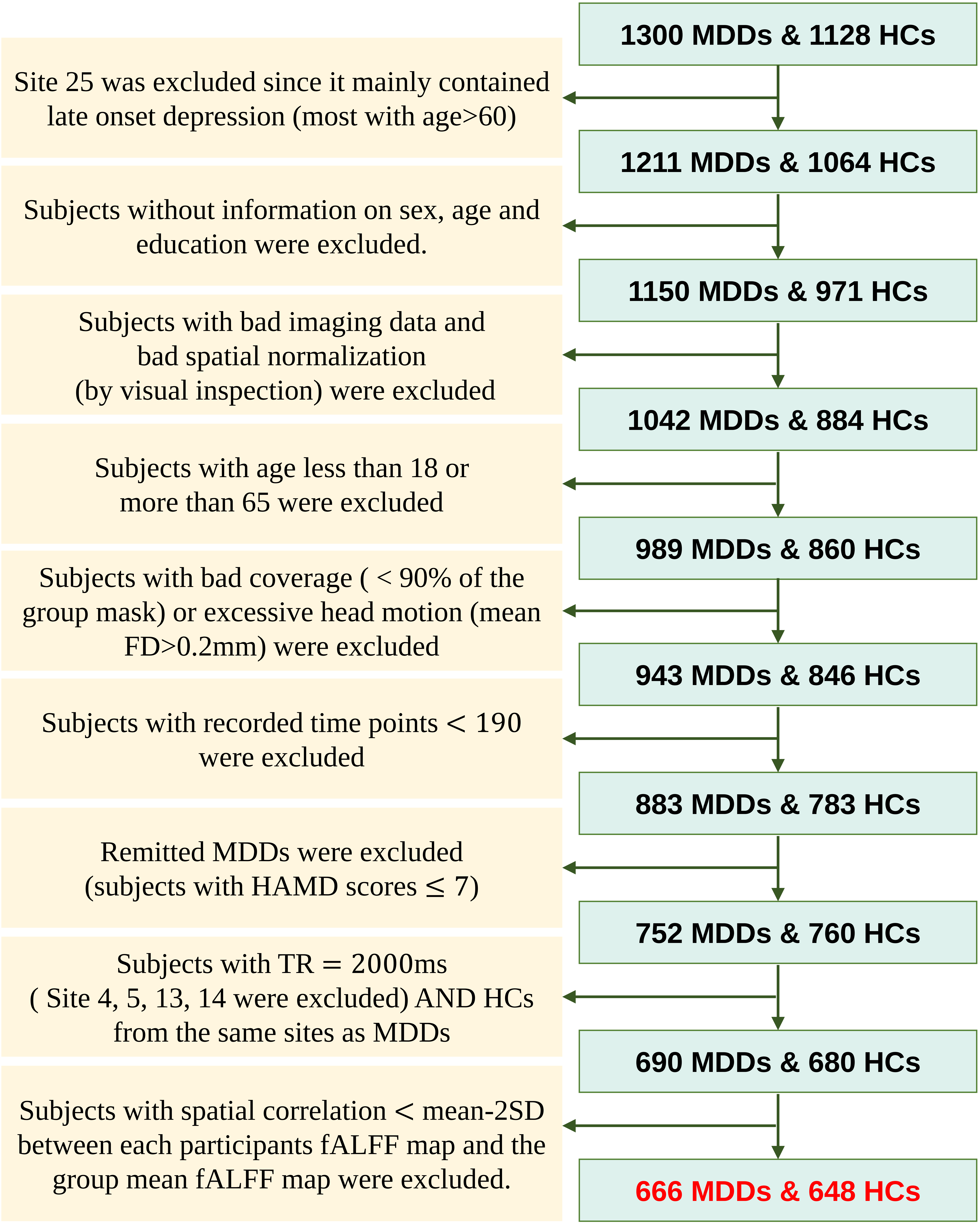
1300 MDDs & 1128 HCs
Site 25 was excluded since it mainly contained late onset depression (most with age>60)
1211 MDDs & 1064 HCs
Subjects without information on sex, age and education were excluded.
1150 MDDs & 971 HCs
Subjects with bad imaging data and bad spatial normalization (by visual inspection) were excluded
1042 MDDs & 884 HCs
Subjects with age less than 18 or more than 65 were excluded
989 MDDs & 860 HCs
Subjects with bad coverage (< 90% of the group mask) or excessive head motion (mean FD>0.2mm) were excluded
943 MDDs & 846 HCs
Subjects with recorded time points < 190 were excluded
883 MDDs & 783 HCs
Remitted MDDs were excluded (subjects with HAMD scores ≤ 7)
752 MDDs & 760 HCs
Subjects with TR = 2000ms (Site 4, 5, 13, 14 were excluded) AND HCs from the same sites as MDDs
690 MDDs & 680 HCs
Subjects with spatial correlation < mean-2SD between each participants fALFF map and the group mean fALFF map were excluded.
666 MDDs & 648 HCs
