# Supplementary material for: Adaptive Whole-Brain Dynamics Predictive Method: Relevancy to Mental Disorders
Source: Research (Wash D C). 2025 Apr 5;8:0648. doi: 10.34133/research.0648 (PMC11971527; doi:10.34133/research.0648)
Supplement: Supplementary 1 — Figs. S1 to S5 Tables S1 to S4 Appendix References [file research.0648.f1.zip › Supplementary Materials.pdf]

Supplementary Materials

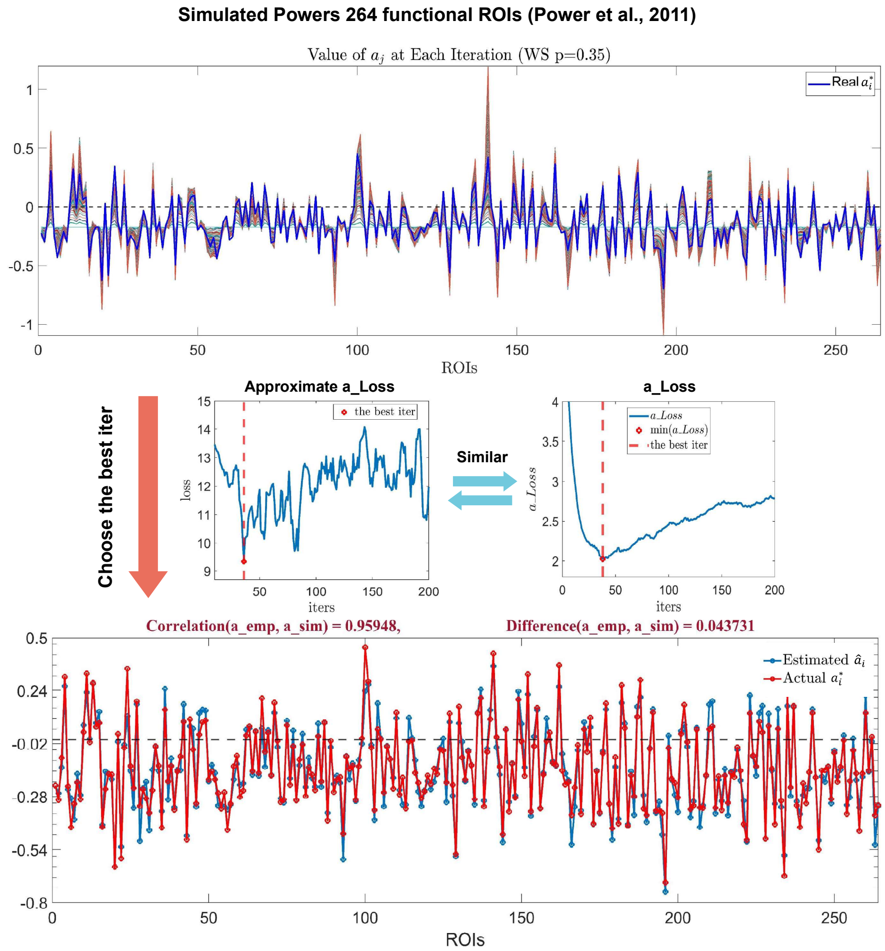

Figure S1: In networks with varying numbers of nodes, the parameter fitting results still demonstrate stability and good performance. This is exemplified by the use of the Power 264 atlas[1].

(a)

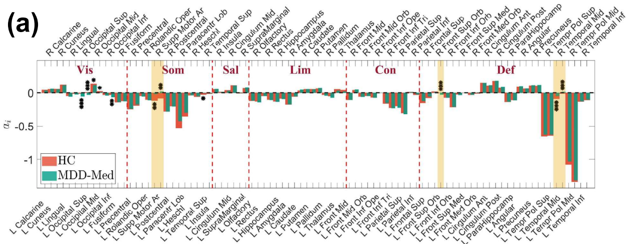

ROIs	Linear-SVM (ACC: 83.81%)	t-test	
	Weight	p	Cohens d
L Temporal Mid	3.6960	$< 0.0001^{***}$	−0.7378
R Supp Motor Ar	2.5668	0.0016^{**}	−0.4384
R Front Sup Orb	−2.3687	0.0010^{**}	0.4614
R Temporal Mid	2.3450	$< 0.0001^{***}$	−0.5980
L Supp Motor Ar	1.8342	0.0017^{**}	−0.4328

(b)

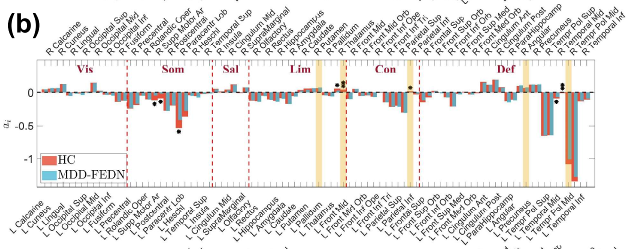

ROIs	Linear-SVM (ACC: 74.33%)	t-test	
	Weight	p	Cohens d
R Angular	2.4063	0.1616	−0.2030
L Parietal Inf	−2.1919	0.0128^{*}	0.3266
R Thalamus	−2.1227	0.0017^{**}	0.4169
R Putamen	1.9184	0.4260	−0.1167
L Tempr Pol Mid	1.7441	0.2218	−0.1825

(c)

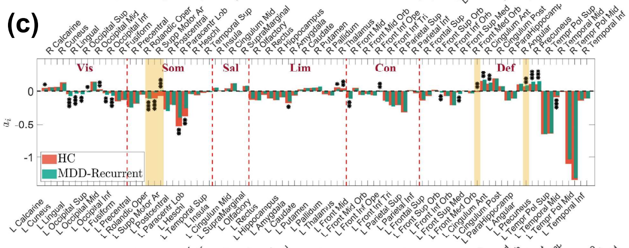

ROIs	Linear-SVM (ACC: 81.75%)	t-test	
	Weight	p	Cohens d
R Angular	2.9172	0.0050^{**}	−0.3245
R Rolandic Oper	−2.6340	0.0004^{***}	0.4165
R Supp Motor Ar	2.2418	0.0007^{***}	−0.3921
R Front Med Orb	2.1749	0.0077^{**}	−0.3034
L Supp Motor Ar	2.0127	0.0004^{***}	−0.4147

(d)

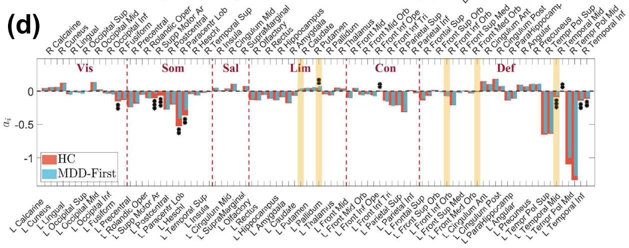

ROIs	Linear-SVM (ACC: 70.66%)	t-test	
	Weight	p	Cohens d
R Front Med Orb	2.7884	0.0848	−0.1590
L Temporal Mid	2.1134	0.0001^{***}	−0.3424
L Caudate	2.0234	0.0221^{*}	−0.2095
R Putamen	1.8667	0.0025^{**}	−0.2680
L Front Inf Orb	−1.8518	0.1579	0.1328

Figure S2: Statistical differences and key ROIs for classification between HC and MDD groups are depicted. In the left figures, ROIs marked with asterisks show significant differences and a medium effect size (t-test: $p < 0.05$, Cohen's $d > 0.25$). Shaded areas represent the top 5 ROIs with the highest weights in the linear-SVM classifier. Right figures show the weights of important nodes and statistical measures (t-test). The shaded areas represent the ROIs with significant weights. (a) HCs vs. 100 medicated MDDs; (b) HCs vs. 159 first episode drug-naïve (FEDN) MDDs; (c) HCs vs. 138 recurrent MDDs; (d) HCs vs. 317 first episode MDDs.

(a)

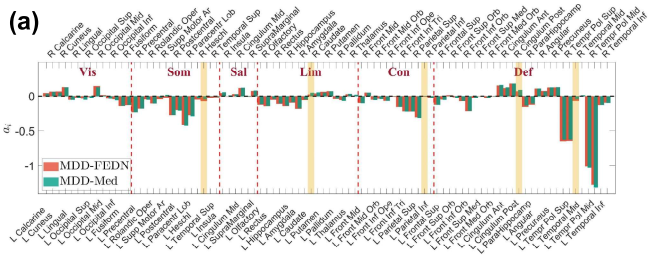

ROIs	Linear-SVM (ACC: 60.06%)	t-test	
	Weight	p	Cohens d
L Temporal Mid	−1.9909	0.2790	0.3548
L Caudate	−1.9797	0.2790	0.3522
L Parietal Inf	−1.9030	0.8310	0.1338
R Heschl	−1.4227	0.6896	0.2446
R Cingulum Post	−1.3616	0.6896	0.1814

(b)

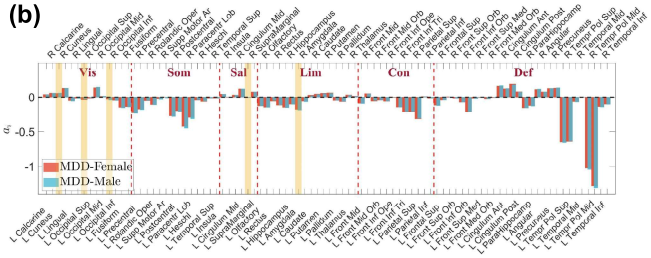

ROIs	Linear-SVM (ACC: 58.20%)	t-test	
	Weight	p	Cohens d
L Occipital Inf	2.4864	0.8312	−0.1812
L Cuneus	−1.8537	0.8312	0.1078
L SupraMarginal	1.7556	0.8312	−0.0905
L Amygdala	1.7544	0.8312	−0.1381
L Occipital Sup	−1.7378	0.8312	0.1173

(c)

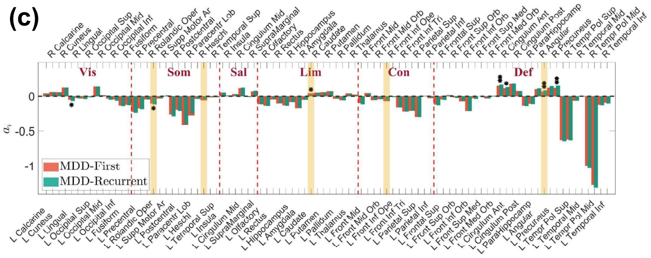

ROIs	Linear-SVM (ACC: 72.09%)	t-test	
	Weight	p	Cohens d
R Heschl	2.5632	0.3208	0.1826
R Angular	−2.4087	0.0040^{**}	0.3968
R Rolandic Oper	−2.0196	0.0180^{*}	−0.3377
L Front Inf Ope	1.8084	0.1937	0.2114
L Caudate	−1.7621	0.0213^{*}	−0.3274

Figure S3: Statistical differences and key ROIs for classification between MDD groups are depicted. In the left figures, ROIs marked with asterisks show significant differences and a medium effect size (t-test: $p < 0.05$, Cohen's $d > 0.25$). Shaded areas represent the top 5 ROIs with the highest weights in the linear-SVM classifier. Right figures show the weights of important nodes and statistical measures (t-test). The shaded areas represent the ROIs with significant weights. (a) 100 first episode medicated (FEMed) MDDs vs. 159 first episode drug-naïve (FEDN) MDDs; (b) 239 male MDDs vs. 427 female MDDs; (c) 138 recurrent MDDs vs. 317 first episode MDDs.

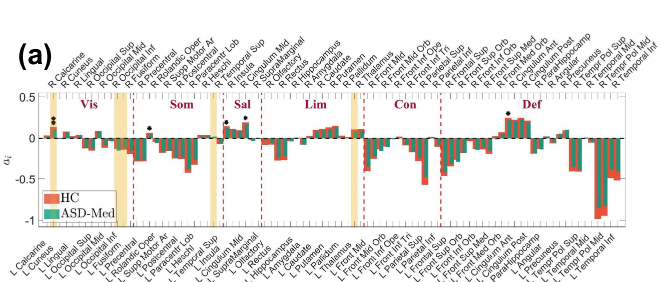

ROIs	Linear-SVM (ACC: 82.88%)	t-test	
	Weight	p	Cohens d
R Calcarine	−3.6806	0.0026**	0.5723
R Occipital Inf	−2.0814	0.5790	0.1324
L Temporal Sup	2.0606	0.6495	−0.0933
L Thalamus	−1.6665	0.0606	0.3786
L Fusiform	1.6511	0.2420	−0.2559

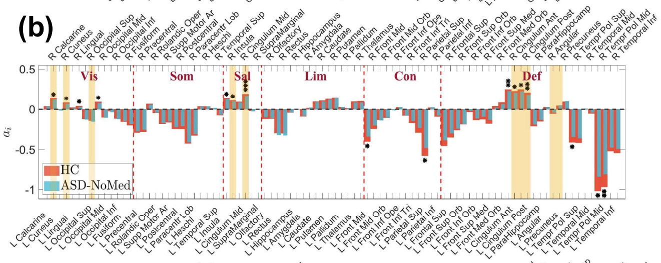

ROIs	Linear-SVM (ACC: 76.11%)	t-test	
	Weight	p	Cohens d
R Occipital Sup	−2.6801	0.5846	0.0823
R Calcarine	−2.1373	0.0295*	0.2873
R Cingulum Post	−2.0344	0.0196*	0.2997
R Cingulum Mid	−1.9130	0.0003***	0.4345
L Precuneus	−1.7917	0.7636	−0.0445
R Angular	1.7122	0.2671	−0.1411
R Cingulum Post	−1.6712	0.0101*	0.3241
R Cuneus	−1.5913	0.0359*	0.2575
R Cingulum Ant	−1.3539	0.0196*	0.2965
R Insula	−1.4634	0.0250*	0.2771

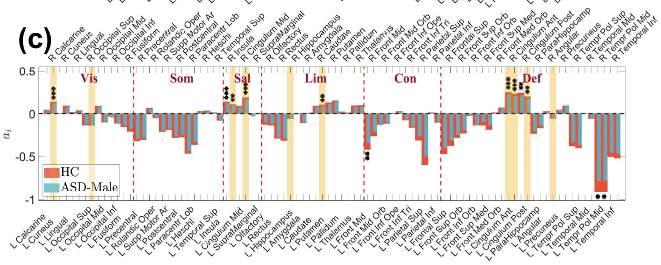

ROIs	Linear-SVM (ACC: 71.54%)	t-test	
	Weight	p	Cohens d
R Occipital Sup	−2.4017	0.9461	0.0054
R Calcarine	−1.9935	0.0006**	0.3365
R Cingulum Post	−1.7965	0.0057**	0.2958
L Hippocampus	1.7929	0.3148	−0.1118
R Cingulum Mid	−1.7562	< 0.0001***	0.4867
R Caudate	−1.7451	0.0064**	0.2711
R Insula	−1.6836	0.0012**	0.3133
L Cingulum Ant	−1.4663	< 0.0001***	0.4109
R Cingulum Ant	−1.2579	0.0001***	0.3854
R Angular	1.2312	0.4809	−0.0807

Figure S4: Statistical differences and key ROIs for classification between HC and ASD groups are depicted. In the left figures, ROIs marked with asterisks show significant differences and a medium effect size (t-test: $p < 0.05$, Cohen's $d > 0.25$). Shaded areas represent the top ROIs with the highest weights in the linear-SVM classifier. Right figures show the weights of important nodes and statistical measures (t-test). The shaded areas represent the ROIs with significant weights. (a) HCs vs. 67 medicated ASDs; (b) HCs vs. 183 non-medicated ASDs; (c) HCs vs. 291 male ASDs.

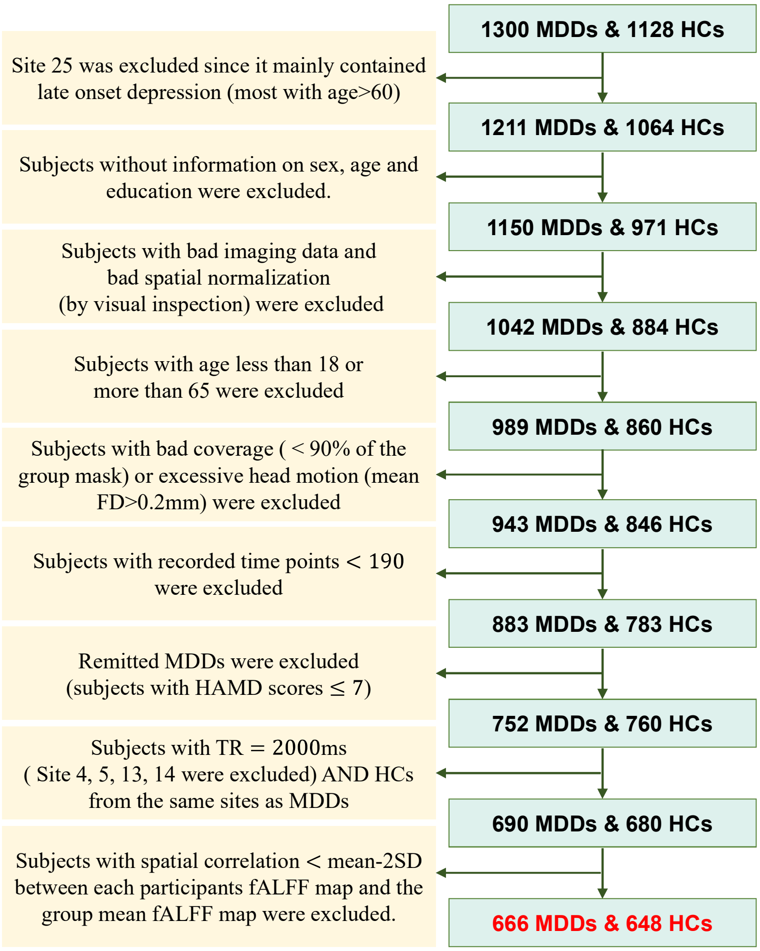

Figure S5: Sample selection

Table S1: The classification performance of MDD-HC.

Contrast	Model	Precision(%)	Recall(%)	F1(%)	Accuracy(%)
MDD FEDN vs HC	linear-SVM	71.26(2.05)	81.66(5.14)	76.03(2.88)	74.33(2.89)
	rbf-SVM	98.79(1.68)	75.83(7.90)	85.54(5.27)	87.47(3.81)
MDD Recurrent vs HC	linear-SVM	78.77(4.08)	87.36(3.81)	82.66(1.15)	81.75(1.46)
	rbf-SVM	90.49(3.58)	96.54(0.90)	93.39(2.29)	93.31(2.02)
MDD First vs HC	linear-SVM	67.12(3.73)	80.68(4.34)	73.22(3.43)	70.66(2.60)
	rbf-SVM	77.26(3.18)	81.52(3.76)	79.19(1.16)	78.59(1.65)
MDD Male vs HC	linear-SVM	65.15(7.39)	61.35(4.56)	63.07(5.25)	64.27(4.62)
	rbf-SVM	68.56(4.94)	60.40(6.97)	64.19(6.11)	66.61(4.37)
MDD Female vs HC	linear-SVM	71.55(5.38)	65.38(6.27)	68.29(5.77)	69.91(4.20)
	rbf-SVM	71.42(2.81)	70.15(2.83)	70.74(2.27)	70.95(3.66)
MDD FEMed vs MDD FEDN	linear-SVM	62.52(9.06)	52.20(4.79)	56.77(6.18)	60.06(6.61)
	rbf-SVM	71.75(8.07)	100.00(0.00)	83.29(5.61)	80.18(6.14)
MDD First vs MDD Recurrent	linear-SVM	71.13(3.23)	74.58(2.90)	72.73(1.66)	72.09(2.04)
	rbf-SVM	100.00(0.00)	76.77(4.07)	86.80(2.58)	88.49(1.83)
MDD Female vs MDD Male	linear-SVM	59.31(5.85)	54.45(3.66)	56.56(3.19)	58.20(3.47)
	rbf-SVM	73.97(3.49)	100.00(0.00)	84.99(2.32)	82.43(2.20)

Table S2: The classification performance of ASD-HC.

Contrast	Model	Precision(%)	Recall(%)	F1(%)	Accuracy(%)
ASD Med vs HC	linear-SVM	76.77(2.44)	94.40(2.13)	84.63(1.39)	82.88(1.76)
	rbf-SVM	94.92(1.66)	97.81(1.38)	96.33(1.28)	96.43(1.25)
ASD NO-Med vs HC	linear-SVM	72.55(2.75)	84.23(5.84)	77.87(3.44)	76.11(3.94)
	rbf-SVM	81.68(4.20)	83.07(3.84)	82.20(1.77)	81.93(2.16)
ASD HandL vs HC	linear-SVM	82.83(16.70)	81.00(11.04)	81.29(12.02)	84.00(8.00)
	rbf-SVM	84.33(13.48)	100.00(0.00)	90.92(7.94)	90.00(9.35)
ASD HandR vs HC	linear-SVM	66.38(3.46)	76.41(6.06)	70.91(3.41)	68.82(2.59)
	rbf-SVM	76.13(6.04)	71.59(5.82)	73.54(4.33)	76.25(4.49)
ASD Male vs HC	linear-SVM	70.35(3.16)	74.61(7.31)	72.18(3.47)	71.54(2.56)
	rbf-SVM	73.61(4.51)	76.52(5.08)	74.82(2.85)	75.02(3.18)
ASD Female vs HC	linear-SVM	76.53(8.86)	82.57(10.03)	79.32(8.91)	79.01(8.79)
	rbf-SVM	85.64(7.67)	91.21(0.37)	88.18(4.06)	87.59(4.32)
ASD non-Med vs ASD Med	linear-SVM	85.39(6.33)	65.88(4.92)	74.09(3.03)	77.05(3.37)
	rbf-SVM	93.25(4.60)	89.33(3.89)	91.17(3.27)	91.08(3.35)
ASD Female vs ASD Male	linear-SVM	82.02(3.61)	91.80(3.25)	86.54(1.88)	85.74(1.94)
	rbf-SVM	99.12(1.08)	100.00(0.00)	99.56(0.54)	99.57(0.53)

Table S3: Comparison of previous studies that used the same REST-meta-MDD dataset for classifying MDD and HC.

Reference	Sample	Sample Size	Brain Parcellation	Feature Selection	Validation Strategy	Classifier	Performance (Accuracy)
Gallo, S et al. (2023) [2]	MDD vs HC	974/974	116 ROIs (AAL atlas)	Functional Connectivity (FC)	5-fold cross-validation strategy	linear-SVM	60.39%
						rbf-SVM	63.15%
						GCN	61.47%
	MDD non med vs HC	255/255				linear-SVM	60.90%
						rbf-SVM	60.87%
						GCN	63.31%
	MDD med vs HC	217/217				linear-SVM	65.15%
						rbf-SVM	65.80%
						GCN	66.50%
	MDD med vs MDD nonmed	183/183				linear-SVM	56.57%
						rbf-SVM	60.99%
						GCN	64.17%
Liu, Q et al. (2023) [3]	MDD vs HC	1276/1103	112 ROIs (Harvard-Oxford atlas)	time series of rs-fMRI data	10-fold cross-validation strategy	Original: a scheme that does not use data augmentation	60.06%
						SDA: a scheme only with spatial data augmentation module	62.22%
						TDA: a scheme only with temporal data augmentation module	62.31%
						STDAC: spatial-temporal data augmentation	63.41%
Dai, P et al. (2022) [4]	MDD vs HC	832/779	116 ROIs (AAL atlas)	Functional Connectivity (FC)	5-fold cross-validation strategy	linear-SVM	61.83%
						Light-GBM	62.94%
				Network Attribute (NA)		linear-SVM	56.67%
						Light-GBM	57.60%
				select 136 specific features (the best performance)		linear-SVM	68.90%
						Light-GBM	67.66%

Reference	Sample	Sample Size	Brain Parcellation	Feature Selection	Validation Strategy	Classifier	Performance (Accuracy)
Zhu, M et al. (2023)[5]	MDD vs HC	830/771	160 ROIs (Dosenbach's atlas)	Functional Fonnectivity (transformed into 0–1 binary adjacency matrices)	10-fold cross-validation strategy	rbf-SVM	59.70%
						Random Forests (RF)	62.30%
						GCN	67.40%
						Deep Graph Convolutional Neural Network (DGCNN)	72.10%
Wang, X et al.[6]	MDD vs HC (Some cross-site classification)	282/251(Site 20); 86/70 (Site 21); 74/74(Site 1)	116 ROIs (AAL atlas)	Functional Fonnectivity (FC)	5-fold cross-validation strategy	Principal Component Analysis (PCA)+SVM	53-54%
						Degree Centrality (DC)+SVM	53-57%
						Eigenvector Centrality (EC)+SVM	53-55%
						Clustering Coefficients (CC)+SVM	54-55%
						Betweenness Centrality (BC)+SVM	52-53%
						CNN (LeCun et al. (1989) [7])	58%
						GCN (Kipf & Welling (2016) [8])	57-58%
						ST-GCN (Gadgil et al. (2020) [9])	60-61%
						No pretext	55-58%
						Unsupervised Contrastive Graph Learning (UCGL) + Window Slicing (the best augmentation strategy)	62-63%

Reference	Sample	Sample Size	Brain Parcellation	Feature Selection	Validation Strategy	Classifier	Performance (Accuracy)
Fang et al. (2023)[10]	MDD vs HC (Some cross-site classification)	282/251(Site 20 as the source domain); 74/74(Site 1 as the target domain)	116 ROIs (AAL atlas)	Use the Functional Fonnectivity (FC) matrix to construct the graph, and input the standardized/normalized ROI-based BOLD signals for each subject	2-fold cross-validation strategy based only on the target data	Node-based brain connectivity features + Linear-SVM (BC-SVM-N)	52.03%
						Graph-based topology features + Linear-SVM (BC-SVM-G)	51.35%
						Node-based + Graph-based features + Linear-SVM (BC-SVM-N-G)	52.03%
						XGBoost (Shi et al., 2021[11])	50.68%
						Multisite domain daptation framework via Low-rank representation (MaLRR (Wang et al., 2019[12]))	54.73%
						Weighted Correlation Kernel based CNN (Wck-CNN (Jie et al., 2020[13]))	53.92%
						Long Short-Term Memory (LSTM (Graves et al., 2012[14]))	51.62%
						Spatial-Temporal convolutional-recurrent neural Network (STNet (Wang et al., 2019))	52.03%
						Domain Adaptation Neuron Network (DANN (Ganin & Lempitsky, 2015[15]))	52.43%
						Discrepancy based Unsupervised fMRI Adaptation Method (UFA-Net)	59.73%

Table S4: Comparison of previous studies that used the same ABIDE dataset for classifying ASD and HC.

Reference	Sample Size	Brain Parcellation	Feature Selection	Validation Strategy	Classifier	Performance (Accuracy)
Heinsfeld et al. (2018)[16]	ASD:505 HC:530	200 ROIs (Craddock 200 atlas)	Functional Connectivity (FC)	10-fold cross-validation strategy	SVM	65.00%
					Random Forests (RF)	63.00%
					DNN	70.00%
Yang et al. (2019)[17]	ASD:505 HC:530	400 ROIs (CC400 atlas)	Functional Connectivity (FC)	5-fold cross-validation strategy	Ridge/ LR/ linear-SVC/ SVC-rbf	71.98/71.79/ 71.40/71.40%
		200 ROIs (CC200 atlas)				69.28/69.09/ 68.60/68.12%
		116 ROIs (AAL atlas)				65.99/66.18/ 65.41/65.60%
		200 ROIs (Harvard-Oxford atlas)				67.92/67.73/ 67.34/67.34%
		116 ROIs (Eickoff-Zilles atlas)				66.09/66.09/ 66.57/65.30%
		110 ROIs (Talairach-Tournoux atlas)				67.54/66.96/ 67.73/66.47%
		160 ROIs (Dosenbach atlas)				63.00/63.19/ 63.48/63.09%
Liu et al. (2020)[18]	ASD:403 HC:468	116 ROIs (AAL atlas)	Dynamic Functional Connectivity (DFC)	10-fold cross-validation strategy	MTFS-L21	71.10%
					M2TFS[19]	73.40%
					MTEN [20]	74.90%
					MTFS-EM: multi-task feature selection method integrating elastic net and manifold regularization	76.80%
Wang et al. (2020)[21]	ASD:419 HC:530	CC200, AAL116 and Dosenbach160	Functional Fonnectivity (FC) feature representa-tions based on three atlases (F_{CC}, F_{AAL}, F_{DOH})	10-fold cross-validation strategy	F_{CCS}	73.39%
					F_{AALS}	73.40%
					F_{DOHS}	69.74%
					$F_{CCS} + F_{AALS}$	73.60%
					$F_{CCS}+F_{AALS}+F_{DOHS}$	76.80%

**Reference**	**Sample Size**	**Brain Parcellation**	**Feature Selection**	**Validation Strategy**	**Classifier**	**Performance (Accuracy)**
Gao et al. (2021)[22]	ASD:419 HC:530	108 ROIs (SRI24 atlas)	the individual-level morphological covariance brain network from the structural MRI	10-fold cross-validation strategy	Auto-encoder (AE)	67.27%
					Random Forests (RF)	53.64%
					SVM	61.82%
					XGBoost (XGB)	60.91%
					Grad-CAM: combine a deep learning classifier and gradient-weighted class activation mapping	71.82%
Ingalhalikar et al. (2021)[23]	ASD:505 HC:530	200 ROIs (Craddock 200 atlas)	Functional Connectivity(FC)	10-fold cross-validation strategy	Random Forests (RF)	60.63%
					Artificial Neural Network (ANN)	71.35%
					Auto-encoder (AE)	69.93%
Deng et al. (2022)[24]	ASD:516 HC:493	200 ROIs (Craddock 200 atlas)	fMRI time series + demographic information	10-fold cross-validation strategy	LSTM	65.68%
					1DCNN	65.43%
					Transformer	66.46%
					Transformer encoder	66.21%
					Transformer decoder	65.63%
					ST-Transformer LA: uses self-attention instead of linear attention	67.25%
					ST-Transformer	68.56%
Yang et al. (2022)[25]	ASD:403 HC:468	444 ROIs (Bootstrap Analysis of Stable Clusters (BASC) atlas)	Functional Connectivity(FC): full correlation	5-fold cross-validation strategy	Logistic Regression (LR)	69.20%
					linear-SVM	68.86%
					kernel-SVM	69.43%
					Deep Neural Network (DNN)	68.45%

Reference	Sample Size	Brain Parcellation	Feature Selection	Validation Strategy	Classifier	Performance (Accuracy)
Kan et al. (2022)[26]	ASD:516 HC:493	200 ROIs (Craddock 200 atlas)	Functional Connectivity(FC)	70% of the datasets for training, 10% for validation and 20% are the test set	Spectral Attention Network (SAN) [27]	65.30%
					Graphormer [28]	60.80%
					VanillaTF	65.20%
					BrainGNN [29]	59.40%
					BrainGB [30]	63.60%
					BrainNetCNN [31]	67.80%
					FBNETGEN [32]	68.00%
					BrainNetGNN [33]	51.20%
					Differentiable Graph Module (DGM) [34]	60.70%
					BRAINNETTF [26]	71.00%
Wen et al. (2023)[35]	ASD:403 HC:468	200 ROIs (Craddock 200 atlas)	fMRI time series + demographic information	10-fold cross-validation strategy	TA-encoder: It is a graph classification model with the proposed topology-aware encoder	66.50%
					BrainGSL-AE: We replace our proposed encoder-decoder framework with vanilla autoencoder for the pretext learning	67.40%
					BrainGSL-GCN: We replace our proposed topology-aware encoder with vanilla GCN for the pretext learning	66.20%
					BrainGSLs: We average the results of 20 different single random BrainGSL models	68.60%
					BrainGSLs-JL: Following the end-to-end training strategy of ASD-DiagNet, BrainGSLs-JL is jointly trained for classification and reconstruction with 10 epochs and then fine-tuned for the classification with extra 50 epochs	68.50%
					BrainGSLs-SRL: We incorporate the signal representation learning module into BrainGSLs	70.40%
					ST-Transformer	71.30%

Appendix References

1. Power JD, Cohen AL, Nelson SM, et al. Functional Network Organization of the Human Brain. Neuron 2011;72:665–78.
2. Gallo S, El-Gazzar A, Zhutovsky P, et al. Functional connectivity signatures of major depressive disorder: machine learning analysis of two multicenter neuroimaging studies. Molecular Psychiatry 2023:1–10.
3. Liu Q, Zhang Y, Guo L, and Wang Z. Spatial-temporal data-augmentation-based functional brain network analysis for brain disorders identification. Frontiers in Neuroscience 2023;17.
4. Dai P, Xiong T, Zhou X, et al. The alterations of brain functional connectivity networks in major depressive disorder detected by machine learning through multisite rs-fMRI data. Behavioural Brain Research 2022;435:114058.
5. Zhu M, Quan Y, and He X. The classification of brain network for major depressive disorder patients based on deep graph convolutional neural network. Frontiers in Human Neuroscience 2023;17.
6. Wang X, Chu Y, Wang Q, et al. Unsupervised contrastive graph learning for resting-state functional MRI analysis and brain disorder detection. Human Brain Mapping 2023;44:5672–92.
7. LeCun Y, Boser B, Denker JS, et al. Backpropagation Applied to Handwritten Zip Code Recognition. Neural Computation 1989;1:541–51.
8. Kipf TN and Welling M. Semi-Supervised Classification with Graph Convolutional Networks. In: *International Conference on Learning Representations*. 2017.
9. Gadgil S, Zhao Q, Adeli E, Pfefferbaum A, Sullivan E, and Pohl K. Spatio-Temporal Graph Convolution for Resting-State fMRI Analysis. Medical image computing and computer-assisted intervention: MICCAI ... International Conference on Medical Image Computing and Computer-Assisted Intervention 2020.
10. Unsupervised cross-domain functional MRI adaptation for automated major depressive disorder identification. Medical Image Analysis 2023;84:102707.
11. Shi Y, Zhang L, Wang Z, et al. Multivariate Machine Learning Analyses in Identification of Major Depressive Disorder Using Resting-State Functional Connectivity: A Multicentral Study. ACS Chemical Neuroscience 2021;12:2878–86.
12. Wang M, Zhang D, Huang J, Yap PT, and Liu M. Identifying Autism Spectrum Disorder With Multi-Site fMRI via Low-Rank Domain Adaptation. IEEE Transactions on Medical Imaging 2019;PP:1–1.
13. Jie B, Liu M, Lian C, Shi F, and Shen D. Designing Weighted Correlation Kernels in Convolutional Neural Networks for Functional Connectivity based Brain Disease Diagnosis. Medical Image Analysis 2020;63:101709.
14. Graves A. Long Short-Term Memory. In: *Supervised Sequence Labelling with Recurrent Neural Networks*. Berlin, Heidelberg: Springer Berlin Heidelberg, 2012:37–45. DOI: 10.1007/978-3-642-24797-2_4.

15. Ganin Y and Lempitsky V. Unsupervised Domain Adaptation by Backpropagation. In: *Proceedings of the 32nd International Conference on Machine Learning*. Ed. by Bach F and Blei D. Vol. 37. 2015:1180–9.

16. Heinsfeld AS, Franco AR, Cameron Craddock R, Buchweitz A, and Meneguzzi F. Identification of autism spectrum disorder using deep learning and the ABIDE dataset. NeuroImage: Clinical 2018;17:16–23.

17. Yang X, Islam MS, and Khaled AMA. Functional connectivity magnetic resonance imaging classification of autism spectrum disorder using the multisite ABIDE dataset. In: *2019 IEEE EMBS International Conference on Biomedical & Health Informatics (BHI)*. 2019:1–4. DOI: 10.1109/BHI.2019.8834653.

18. Liu J, Sheng Y, Lan W, Guo R, and Wang J. Improved ASD Classification using Dynamic Functional Connectivity and Multi-task Feature Selection. Pattern Recognition Letters 2020;138.

19. Biao. J, Daoqiang. Z, Bo. C, and Dinggang S. Manifold regularized multitask feature learning for multimodality disease classification. Human Brain Mapping 2014;36.

20. Zou B, Lampos V, and Cox I. Multi-Task Learning Improves Disease Models from Web Search. In: *the 2018 World Wide Web Conference*. 2018.

21. Wang Y, Wang J, Wu FX, Hayrat R, and Liu J. AIMAFE: Autism spectrum disorder identification with multi-atlas deep feature representation and ensemble learning. Journal of Neuroscience Methods 2020;343:108840.

22. Gao J, Chen M, Li Y, et al. Multisite Autism Spectrum Disorder Classification Using Convolutional Neural Network Classifier and Individual Morphological Brain Networks. Frontiers in Neuroscience 2021;14:629630.

23. Ingalhalikar M, Shinde S, Karmarkar A, Rajan A, Rangaprakash D, and Deshpande G. Functional Connectivity-Based Prediction of Autism on Site Harmonized ABIDE Dataset. IEEE Transactions on Biomedical Engineering 2021;68:3628–37.

24. Deng X, Zhang J, Liu R, and Liu K. Classifying ASD based on time-series fMRI using spatial-temporal transformer. Computers in Biology and Medicine 2022;151.

25. Yang X, Zhang N, and Schrader P. A study of brain networks for autism spectrum disorder classification using resting-state functional connectivity. Machine Learning with Applications 2022;8:100290.

26. Xuan K, Wei D, Hejie C, Zilong Z, Ying G, and Carl Y. BRAIN NETWORK TRANSFORMER. In: *Advances in Neural Information Processing Systems*. 2022.

27. Kreuzer D, Beaini D, Hamilton WL, Létourneau V, and Tossou P. Rethinking Graph Transformers with Spectral Attention. In: *Neural Information Processing Systems*. 2021.

28. Ying C, Cai T, Luo S, et al. Do Transformers Really Perform Badly for Graph Representation? In: *Advances in Neural Information Processing Systems*. Vol. 34. 2021:28877–88.

29. Li X, Zhou Y, Dvornek N, et al. BrainGNN: Interpretable Brain Graph Neural Network for fMRI Analysis. Medical Image Analysis 2021;74:102233–.

30. Cui H, Dai W, Zhu Y, et al. BrainGB: A Benchmark for Brain Network Analysis With Graph Neural Networks. IEEE Transactions on Medical Imaging 2023;42:493–506.

31. Kawahara J, Brown CJ, Miller S, Booth BG, and Hamarneh G. BrainNetCNN: Artificial Convolutional Neural Networks for Connectomes. In: *Annual Neuroscience Extravaganza, Centre for Brain Health, BC, Canada.* 2016.

32. Kan X, Cui H, Lukemire J, Guo Y, and Yang C. FBNETGEN: Task-aware GNN-based fMRI Analysis via Functional Brain Network Generation. In: *Proceedings of Machine Learning Research.* Vol. 172. 2022:618–37. URL: https://proceedings.mlr.press/v172/kan22a.html.

33. Mahmood U, Fu Z, Calhoun VD, and Plis S. A Deep Learning Model for Data-Driven Discovery of Functional Connectivity. Algorithms 2021;14:75.

34. Kazi A, Cosmo L, Ahmadi SA, Navab N, and Bronstein MM. Differentiable Graph Module (DGM) for Graph Convolutional Networks. IEEE Transactions on Pattern Analysis and Machine Intelligence 2023;45:1606–17.

35. Wen G, Cao P, Liu L, et al. Graph Self-Supervised Learning With Application to Brain Networks Analysis. IEEE Journal of Biomedical and Health Informatics 2023;27:4154–65.
